# Cancer Stem Cell Theory and the Warburg Effect, Two Sides of the Same Coin?

**DOI:** 10.3390/ijms15058893

**Published:** 2014-05-19

**Authors:** Nicola Pacini, Fabio Borziani

**Affiliations:** Laboratorio Privato di Biochimica F. Pacini, via trabocchetto 10, 89126 Reggio Calabria, Italy; E-Mail: fabioborziani@tin.it

**Keywords:** cancer, glycolysis, cancer stem cells, Warburg effect, ATP, mitochondria, epigenetic modulation, oxidative phosphorylation, differentiation, aerobic glycolysis, entropy, free energy, dissipative structure, epithelial-mesenchymal transition, cancerogenesis, human embryonic stem cells, induced pluripotent stem cells, self renewal

## Abstract

Over the last 100 years, many studies have been performed to determine the biochemical and histopathological phenomena that mark the origin of neoplasms. At the end of the last century, the leading paradigm, which is currently well rooted, considered the origin of neoplasms to be a set of genetic and/or epigenetic mutations, stochastic and independent in a single cell, or rather, a stochastic monoclonal pattern. However, in the last 20 years, two important areas of research have underlined numerous limitations and incongruities of this pattern, the hypothesis of the so-called cancer stem cell theory and a revaluation of several alterations in metabolic networks that are typical of the neoplastic cell, the so-called Warburg effect. Even if this specific “metabolic sign” has been known for more than 85 years, only in the last few years has it been given more attention; therefore, the so-called Warburg hypothesis has been used in multiple and independent surveys. Based on an accurate analysis of a series of considerations and of biophysical thermodynamic events in the literature, we will demonstrate a homogeneous pattern of the cancer stem cell theory, of the Warburg hypothesis and of the stochastic monoclonal pattern; this pattern could contribute considerably as the first basis of the development of a new uniform theory on the origin of neoplasms. Thus, a new possible epistemological paradigm is represented; this paradigm considers the Warburg effect as a specific “metabolic sign” reflecting the stem origin of the neoplastic cell, where, in this specific metabolic order, an essential reason for the genetic instability that is intrinsic to the neoplastic cell is defined.

## 1. Introduction

The origin of neoplasms has always been an important aim of researches and surveys. In 1937 Jacob and Morton indicated that a single leukemic cell when transplanted in a mouse reproduced the disease in all respects [1]. Moreover, the monoclonal origin of antibodies in multiple myeloma among other facts suggests the monoclonal origin of neoplasms, whereas since the 70s and later in the 80s, the paradigm was asserted that the origin of neoplastic transformation first and the promotion phase second were to be researched in the establishment of multiple, unconnected and causal genetic mutations. During a later stage, thanks also to the works of the last 30 years, epigenetic alterations and changes in the methylation pattern and in chromatin remodeling support multiple genetic alterations. Thus, at the end of the 20th century, the leading paradigm of the origin of neoplasms was the stochastic model or rather somatic cells as a consequence of unconnected, causal genetic and epigenetic mutation assembly, leading to the processes of transformation, promotion, development and metastasis, marking all of the occurrences of a neoplasm.

However, the above-mentioned pattern, although describing coherently several aspects of the neoplastic process, such as the carcinogenesis from mutagens agents, leaves many issues unresolved. In fact, in the *in vivo* xenograft tumor model, many cells are required to activate the development of a neoplasm, thereby negating the monoclonal hypothesis, as it neither considers nor explains the morphological-functional diversity and the heterogeneity of the various neoplastic appearances, which, although often discerning mutations in common genes, show peculiarity and a typical morbidity. However, a contrasting situation can also arise, for example, the p53 gene neither is altered in each cancer nor is its alteration essential for the development of a neoplasm. In addition, cancers with identical isotypes often have different mutations and a similar clinical progress [2–7].

Furthermore, it is interesting to underline how, even during the period of embryonic and postnatal formation, some neoplastic appearances develop and do not lead to a clinically relevant neoplasm; for example, Mori *et al.* have highlighted that pediatric acute leukemia and chromosomal translocation develop with a frequency of greater than 100% in infants compared to the pediatric population during the postnatal period, who later do not develop a clear disease but live a normal psycho-physical growth pattern [8]. These results contrast with the monoclonal hypothesis [9].

In recent years, the hypothesis of the so-called cancer stem cell (CSC) theory progressed, beginning in the 90s when Lapidot *et al.* demonstrated that only a small population of leukemic cancer cells with the CD34+CD38− phenotype, could cause cancer after being inoculated in a non-obese diabetic/severe combined immune deficient mouse [10]. Later, it was illustrated and demonstrated that this breeding was a matter of neoplastic stem cells [11].

Since this time, similar results have been achieved for mammary neoplasms [12], cerebral neoplasms [13,14], pulmonary neoplasms [15], colon neoplasms [16], ovary neoplasms [17,18], pancreas neoplasms [19,20], prostate neoplasms [21] and thyroid neoplasms [22], in addition to many additional types of neoplasms, both solid and belonging to the immune-hematopoietic system [23]. It has also been underlined that precisely this under-population supports the cancer growth and causes chemoresistance [24–26].

**Figure 1.**
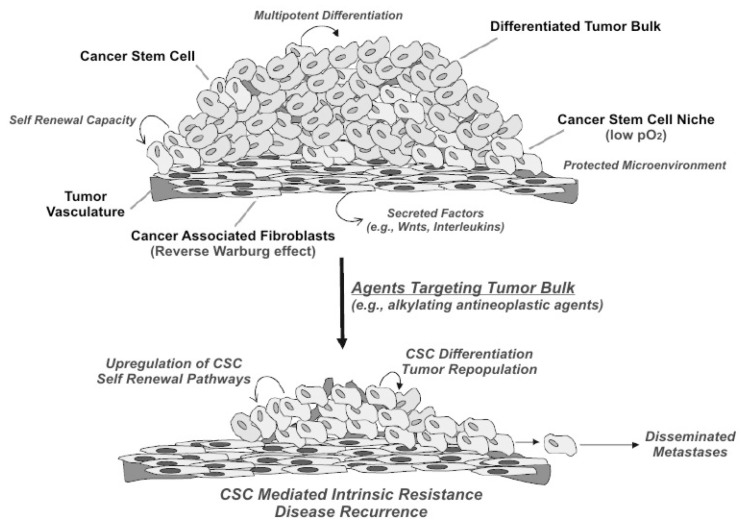
Some important features of the cancer stem cell (CSC) theory are highlighted, the neoplastic tissue, as the normal tissue, is carried and renewed by its stem cell population, which self-renews; CSCs, similar to other stem cells, exist in niches with a suitable microenvironment of low pO_2_; similar to normal tissue, the neoplastic tissue interactions with cells in the microenvironment are fundamental; and traditional cytostatic drugs, such as alkylating agents, are active in the neoplastic bunk but less in CSCs. From this consideration will result a potential mechanism of metastasization. (Modified from Curtin and Lorenzi [24])

Even though this pattern, which is summarized in Figure 1 (modified from Curtin and Lorenzi [24]), was already highlighted in the 70s and was considered efficient in the case of teratoma, this event was found to be an exception to the general rule of the monoclonal stochastic pattern [27].

The two patterns, the CSCs pattern and the monoclonal stochastic pattern, are neither mutually exclusive nor necessarily completely contradictory [27–29], stem cells are in the G0 phase of the cell cycle for long periods, and because of their longevity, stem cells are particularly subject to mutations and genetic/epigenetic alterations regarding the origin of CSCs. Many authors have stated that these cells can originate genetic or epigenetic mutations that are generated by carcinogenic agents or because of the deregulation of various pathways within the recesses of their own microenvironment. Moreover, a genetic mutation or an epigenetic change, depending on the stem cell, is particularly dangerous because it is inherited and passed on to the daughter cells. Another possible hypothesis, even if it still requires definition, regards a somatic cell, which, as a result of some genetic/epigenetic events and in the modulation of the warning network, can be redesigned and return to the stem section to obtain again the typical morphological and functional features [27].

Stem cells, including neoplastic stem cells, have many features in common, the ability of self-renewal, the competence of differentiation, the active expression of the telomerase, the activation of anti-apoptotic pathways, the increase of all of the active membrane transports, and the ability of metastasis migration [30].

Zygotes and human embryonic stem cells (hESCs) and those of other species, which are adult stem cells with reduced differentiating potential (that play an essential role in the cell turnover of adult individuals), have some common features, they live in recesses; are affected by clear conditions that are connected to the microenvironment; show asymmetric mitosis; and have a typical metabolic structure with a lowered oxidative phosphorylation, high glycolysis and reduced levels of intracellular ATP levels and reactive oxygen species (ROS) [31–33].

This specific metabolic structure has been verified in adult stem cells with reduced differentiation potential and in pluripotent embryonic stem cells, by now this circumstance has been clarified through vast material and independent studies. Similarly, it has been demonstrated by that passing from an undifferentiated stem state to a superior differentiation state results in the recovery of the oxidative phosphorylation and of mitochondrial biogenesis, as well as an increase in the ROS production. This result has been demonstrated in hESCs, in myotubes and in myoblasts during their differentiation in muscle tissue in mesenchymal cells during the differentiation in osteoblasts [34,35]. Recently, Stringari *et al.* and Wright *et al.*, through the development of a technique to measure the NADH/NAD^+^ ratio in living tissues, highlighted a glycolytic gradient in the little intestines of rats under physiological conditions [36,37]; this gradient decreases beginning in the intestinal stem cells up to the differentiated erythrocytes, decreasing in glycolytic action, increasing gradually as cellular differentiation progresses.

In addition to mammals, a close dependency between undifferentiated state and glycolytic phenotype has also been studied in many invertebrates, such as in neurogenesis in Drosophila melanogaster [38] and even in Caenorhabditis elegans, in the lower vertebrates, and in non-mammalian vertebrates. For example, during the maturation of oocytes in amphibians, the metabolism is typically glycolytic, and the tricarboxylic acid cycle is maintained primarily by glutamine, whereas oxidative phosphorylation is strongly depressed to gradually increase later through development and maturation [39]. A similar phenomenon can be noticed during the development of rat embryos [40], this is a biological constant that is common to every species.

In recent years, research of stem cells has advanced considerably. Another meaningful step has been the discovery of the so-called induced pluripotent stem cells (iPSCs), which are somatic cells that, by means of the modulation of a complex network of growth factors and genes, are reprogrammed in the stem cell-like state [41,42]. From a metabolic perspective, it is interesting to note that, when passing from somatic cells to iPSCs, beyond all genetic or epigenetic modifications, the basic conditions for reprogramming are a new modulation of the mitochondrial work and a transit from an oxidative phosphorylation state to a merely glycolytic metabolism [43]. Furthermore, Vazquez-Martin *et al.* have demonstrated that the mitochondrial H^+^/ATP synthase is down-regulated and that various inhibitory factors are activated in addition to a sharp change in the metabolism of fatty acids [44]. The suppression and/or the reactivation of these changes in H^+^/ATP synthase strongly reduce or stop the induction process from a somatic cell to an iPSC. There is also a sharp change in the morphology of mitochondria during the reprogramming process [45–47]. The arrest of the oxidative phosphorylation and of the transport chain of electrons is connected to the retention of the undifferentiated state [48]. Moreover, a close connection among the mitochondrial functions, glycolytic phenotype and differentiation has been highlighted as a result of the following event, hexokinase II inhibitors and glycolysis inhibition through other means deeply reduce or totally suppress the reprogramming process according to the degree of metabolic inhibition [45,49,50].

Even though in hESCs, iPSCs, adult stem cells and cancerous human embryonic cells there are some differences in the subtle metabolic signs, we can conclude that the undifferentiated state, similar to neoplastic tissue, is characterized by a decrease in oxidative phosphorylation, a reduced level of intracellular ATP and a smaller production of ROS relative to the differentiated state [43,44,48,51,52]. These data have been confirmed recently in human osteosarcoma cells [53]. Thus, another relevant and interesting perspective is that of the so-called epithelial-mesenchymal transition, which is commonly observed in several neoplasms of epithelial origin during the metastatization process [54,55], further illustrating phenotypic dedifferentiating reprogramming.

Furthermore, the glycolytic phenotype of stem cells certainly recalls the main feature that is almost universal of each neoplastic cell, the Warburg effect, which is the marked dependence for the energy supply from glycolysis [56].

## 2. The Warburg Effect

It has been known for more than 85 years that almost all neoplastic cells, of an epithelial or mesenchymal nature, show a deep alteration of their metabolic works and, in particular, a marked shift towards the glycolysis with a pronounced decrease in the mitochondrial functions or rather of oxidative phosphorylation. This phenomenon is called the “Warburg effect” and takes its name from Otto Heinrich Warburg, the researcher who first described this peculiarity [57].

Glycolysis symbolizes one of the first bio-energetic mechanisms to appear during the eukaryotic phylogeny. Glycolysis leads to the oxidation of glucose with the formation of compounds with three carbon atoms. This metabolic pathway is anaerobic; however, in neoplastic cells, glycolysis is often present independently of O_2_. Therefore, we are dealing with aerobic glycolysis [58].

Historically, Warburg in the mid-50s asserted that aerobic glycolysis could not be a common pathogenetic feature of neoplastic cells but a basic etiologic mechanism of neoplasms [59]. This hypothesis was discussed and opposed for many years. However, in recent years, an increasing number of independent studies and researchers have focused their attention on this phenomenon and on the sets of metabolic changes arising in neoplastic cells and have shown that, similar to dealing with genes and epigenetic factors of warning cells such as growth factors, cytokines and so on, the neoplastic process is marked by a vast alteration of its energetic metabolism [60].

At first, it was believed that the greater glycolysis rate was caused by the lack of vascularization that inherently involved a lower local oxygenation and could explain the glycolytic switch as a local homeostatic response to the lesser pO_2_. However, many researchers have highlighted that this phenomenon is hardly affected by vascularization and local perfusion conditions; *i.e.*, it is conditioned but it is primarily independent [61,62].

Another aspect that has been discussed in recent years, an aspect that caused much disapproval of Warburg and his colleagues’ work, regards a strong glycolytic switch that is often present in neoplastic tissues; this switch is independent of pO_2_ but the oxygen consumption is not at all suppressed or, rather, there is not always a reduction in the oxidative O_2_-dependent phenomenon. This occurrence was explained on the basis of the theory of Lynen’s, who was the first to theorize that the first cause of the Warburg effect was not the cellular inability for using O_2_ in the usual oxidizing-reducing processes but an uncoupling of oxidative phosphorylation which could also be incidental to the etiopathogenesis of neoplasms [63].

The peculiarities of energy metabolism in cancer cells are imputable not only to a markedly glycolytic phenotype, but also to some essential features such as a heavy imbalance in the NADH/NAD^+^ ratio with a marked production of lactate and reduction of pyruvate into the Krebs cycle to maintain the high glycolytic flux and prevent an exaggerated store of NADH and a marked production of lactic acid [64–66]. The produced lactic acid, as a consequence the pH decrease, promotes neoangiogenesis through hypoxia-inducible factor-1 (HIF-1) stimulation from endothelial cells, connective tissue degradation, the arrest of T cells chemotaxis and metalloproteinases activation [67–71]. Furthermore, for example, the overexpression of the lactate dehydrogenase-5 (LDH-5) isoform is a well-known fact in the great majority of human cancers [72–74]. A remarkable proliferative action is carried out by LDH-5 inhibition both *in vitro* and *in vivo* [65,75].

However, the general framework of the Warburg effect is more complex because there is not only a marked increase of glycolysis and its deregulation compared with normally glycolytic cells, but also of glutamine and citrate catabolism aberration with several consequences. Above all, there are many disturbances in the electron transport chain and several alterations of the mitochondrial function. In many neoplastic cells there is an overexpression of NADPH/NADH-oxidase (NOX), which is a membrane enzyme that is connected to several protein structures and is regulated by the rat sarcoma oncogene [76,77]. The activity of these enzymes is considerably increased in cancer cells and leads to the formation of large amounts of superoxide anion and hydrogen peroxide. Recently, Lu *et al.* demonstrated that NOX activity both *in vivo* and *in vitro* is necessary for the maintenance of the strong glycolytic flux as it adjusts the alterations of NADH/NAD^+^ ratio [78]; NOX activity also contributes to increasing the ROS, which is typical of cancer cells. Recently, Lin *et al.* demonstrated that the epithelial-mesenchymal transition *in vitro* and *in vivo* is connected to the origin of Warburg effect and to citrate synthase deregulation [79].

Moreover, there is a connection in several cellular lines *in vitro* and *in vivo* among malignancy, epithelial-mesenchymal transition and intracellular ATP levels; apart from this connection, the reactivation of a suitable ATP level activates again p53 and suppresses the epithelial-mesenchymal transition.

In addition, a correlation between the Warburg effect and neoplastic transformation or its modifications has also been suggested after traditional tests of transformation in the embryos of chicken with Rous sarcoma virus; such a transformation is connected to a clear glycolytic shift [80]. Similarly, Darekar *et al.* demonstrated that the transformation of B lymphocytes and their immortalization as a consequence of the Epstein-Barr virus is closely related to the glycolytic phenotype of the Warburg effect [81].

## 3. Partial Pressure of O_2_

As previously noted, the Warburg effect is related to the lesser local oxygenation, but it has been repeatedly highlighted that even under conditions of good oxygen and nutrient contribution, this phenomenon is not modified. It has been known for more than 40 years that neoplastic cells grow, renew themselves and reproduce best way in a hypoxic setting [82]; for example, human melanomas, plated and grown in cultivation, develop better under hypoxic conditions [83]. Moreover, a low pO_2_ is closely related to the maintenance of an undifferentiated state in human embryonic cells [84]. The same hypoxia fosters the remodeling of somatic cells to iPSCs [85].

In recent years, researchers have studied angiogenesis inhibition as a possible target therapy. Normally the tissues under physiological conditions are exposed to a pO_2_ quantity of 20 mmHg, whereas in neoplastic tissue, there is a marked reduction that is variable with the sizes of the lesion, on average approximately ≤5 mmHg. Strongly hypoxic cancers, sarcomas and/or carcinomas are correlated with a worse prognosis and a marked increase in metastasization potential [86–90]. In addition, the cancer hypoxic setting, *in vivo* and for various neoplastic types, promotes the metastasization process [91–96]. Recently, Ebos *et al.* demonstrated that treatment with powerful and effective angiogenesis inhibitors enhances metastasization [97]. If angiogenesis inhibition is in theory useful in neoplasms treatments, there is an inconsistency between the pO_2_ effect and the angiogenesis arrest. However, if we consider that the majority of neoplastic cells use glycolysis as a sustenance mechanism, then it is interesting to note that, even if the glycolysis produces only 2 molecules of ATP from a glucose molecule (compared to the 32 molecules that are produced by oxidative phosphorylation), under conditions of intense metabolic activity, the glycolytic flux can increase and maintain constant ATP levels if an adequate contribution of nutrients (glucose and glutamine) is maintained [98,99]. It is also interesting to note that the vascular system within the neoplastic tissue is chaotic and lacking in efficiency, there is arteriovenous anastomosis, the non-striated musculature is absent, the permeability is extremely high and exchanges are reduced [100].

## 4. Genetics, Epigenetics and Metabolism

According to the classical stochastic model, neoplasms are the product of accumulations of mutations at the genetic and epigenetic levels. However, as already discussed in the beginning of this report, there are some inconsistencies that are not explained or characterized by this model. For example, if a neoplasm could be caused by a single mutation, considering that in the adult there are approximately 1 × 10^12^ cell divisions a day, and if we consider that the rate of mutation for cell division is 1.1 × 10^−8^, there will be 1 × 10^4^ mutations a day, and the incidence of tumors will be very high. If we consider 4–6 independent point simultaneous mutations, for example, 5 for the neoplastic transformation, we will have (1 × 10^12^) × (1.1 × 10^−8^)^5^ = 1.61 × 10^−28^ chances every day; assuming an unlikely life span of 120 years, we would have (120 × 365 × 10^12^) × (1.1 × 10^−8^)^5^ = 7.05 × 10^−24^ chances, meaning that neoplasms would occur with a probability close to 0 [7,101–103]. In addition, if we consider that some mutations and/or epigenetic silencing events are common to several types of malignancies and if we admit that only a few mutations and/or silencing events are necessary, the possibility of the simultaneous development of two neoplastic forms in the same subject will be equal to 0.

In addition, many epigenetic alterations are interdependent from metabolic abnormalities rather than being provoked by these abnormalities and are not caused by them, the high production of lactate causes a significant imbalance in the NADH/NAD^+^ ratio that can modulate the level of histone acetylation through interaction with proteins of the sirtuins family [104–106]; the latter mechanism has also recently been well highlighted in human glioma cells [107]. Similarly, in neoplastic cells occurs a strong imbalance in the ratio between reduced glutathione and oxidized glutathione, where large amounts of S-adenosylmethionine (SAME) are used to increase the ratio, causing severe and complex repercussions on the methylation level of many genes [104,105,108,109]. In turn, the metabolism of SAME can interfere with the NADH/NAD^+^ ratio through serious consequences, as have been well documented in alcohol-mediated carcinogenesis [110]. One of the enzymes responsible for the Warburg effect, the M2 isoform of pyruvate kinase, modifies and regulates the expression of several genes that are involved in the cell cycle, directly phosphorylating histone H3 [111]. The nuclear translocation of hexokinase II in cancer cells of the uterine cervix are involved in the mechanisms of gene expression regulation [112]. With regard to this situation, the nuclear translocation of hexokinase-II was demonstrated in the yeast Saccharomyces cerevisiae and regulates and modulates the activity of many glycolytic enzymes [113]. This yeast is a phylogenetically ancient organism that is particularly suitable for studies of the relationship between energy metabolism and cell growth and partly also to the study of some common characteristics of neoplasms. Friis *et al.* have shown that the glycolytic rate and its speed in the unit of time in this unicellular organism directly influence the acetylation of a large number of histones and are directly connected to the differential expression of numerous genes [114]. In addition, Daran-Lapujade *et al.* have demonstrated that the flow through the glycolytic pathway produces modifications in development, in growth and in the cell cycle without considering genetic factors, and it is evident that there is a hierarchically greater contribution of the metabolome compared to the processes of gene expression [115].

As shown by Chen *et al.*, it is also important to note that in the yeast Saccharomyces cerevisiae the DNA replication phase or S phase of the cell cycle is dependent on glycolysis and is closely related to the activity of metabolic networks rather than to genetic and epigenetic factors [116]. This phenomenon acquires an important and functional meaning because during the S phase of the cell cycle, there is a reduction in the level of free radicals and ROS, indicating a greater preservation of genomic integrity.

However, there is another important mechanism through which the energy metabolism can adjust and modulate the processes of damage-repair and replication and synthesis of DNA. In fact, an inhibition of oxidative phosphorylation in murine leukemic cells and cervical cancer cells can inhibit and negatively interfere with the processes of DNA damage and repair [117,118]. In addition, the levels of intracytoplasmic and nuclear ATP levels are essential for DNA repair and that respiratory depression, through 2,4-dinitrophenol, blocks or abolishes the repair processes of DNA that are induced by gamma radiation [119,120].

A close link between cellular respiration and ATP levels for efficient DNA repair was also noticed in strains of Saccharomyces cerevisiae during damage and repair processes [121]. In prokaryotes, as well as in strains of *Escherichia coli*, the ATP/ADP ratio is essential for DNA damage, repair and recombination [122]; however, for the efficient repair of genotoxic damages, an efficient system of ATP production is required [123]. Similarly, it was well documented that for a good response and repair of the damage, particularly during the earliest stages of genotoxic damage, somatic cells of invertebrates and mammals respond with a strong production of ATP.

Another interesting fact is the relationship between the ATP/ADP ratio and the S phase of the cell cycle. Rapaport *et al.* demonstrated in murine fibroblasts a close relationship between the S phase of the cell cycle and the nuclear and cytoplasmic ATP/ADP ratio, showing that during the S phase, there is a strong reduction of the ATP/ADP ratio, both at the nuclear and cytoplasmic levels [124]. Recently, similar results have also been found in strains of *Escherichia coli*, where the ATP/ADP ratio decreases drastically during the S phase of the cell cycle [125]. It is also interesting to consider that in the metabolism of nucleic acids, at the level of the cell nucleus participate dozens of enzymes, the vast majority being ATP dependent [126,127]. This behavior differs completely from that of many glycolytic enzymes, which couple the energy of ATP hydrolysis to favor endergonic reactions. Many enzymes that govern the remodeling of DNA and RNA use ATP even for exergonic reactions that are thermodynamically favorable [127]. This result is related to the fact that ATP hydrolysis is essential to ensure the stability of the entire genome also in processes that are not strictly dependent on broad energy restoration.

Similarly, the efficiency and activity of the protein p53 is directly influenced by the ATP/ADP ratio; increasing ADP levels causes a clear inactivation of this protein, affecting its ability to interact with DNA [128]. In addition, the ATP/ADP ratio is essential for all of the processes of chromatin remodeling, and its close dependence has been highlighted by several independent authors [129,130]. In conclusion, an optimal ATP/ADP ratio and an efficient production of ATP are essential for the proper metabolism of nucleic acids [126,127].

It should also be noted that there is an important relationship between the ATP reserves and the efficiency of the repair process from various genotoxic damage [119,120,131,132]. Several authors have recently confirmed that both stem cells and iPSCs show a greater susceptibility to genetic damage and a smaller efficiency in the processes of DNA damage repair [133–136]. This instability appears constant and is inversely proportional to cellular differentiation, as has been established in previous studies [137–139].

In the light of all of the expressed considerations and of those that will be described in the section discussing mitochondria, we can hypothesize a connection between the normal respiratory activity and the genetic stability, even with DNA repair processes. Stem cells and iPSCs, although in relation to various genotoxic damages, have more efficient repair systems and are characterized by a greater instability, as previously highlighted by several authors in different contexts [140].

Moreover, independent of the ATP levels, hESCs and other adult stem cells show greater sensitivity to genotoxic damage, as Nouspikel and Cousin *et al.* recently demonstrated [136,141]. In addition, it should be highlighted that several carcinogenic agents stimulate the induction of HIF-1; they also cause the over-expression of various glycolytic enzymes.

## 5. Population Dynamics and Microenvironment

The new concepts emerging from CSC theory highlight another essential idea, a neoplasm is not composed of only one type of cell but of a heterogeneous population of normal somatic cells (fibroblasts, mast cells, *etc.*), CSCs and differentiated cells, even though aberrant and/or in the process of differentiation toward a clinical cancer. Therefore, from a biological perspective, it is possible to speak about the cell population dynamics, and many scholars have applied to these dynamics the concept of Darwinian fitness.

It is important to remember that a model known as the reverse Warburg Effect has recently been proposed, in which, as validated in breast cancer, there is a very strong interaction between the stromal cells and the myofibroblasts [142]. This particular phenotype originates in the process of wound healing under the action of TGF-beta and is involved in numerous processes, both physiological and pathophysiological. In addition, it has been shown that in breast tumors, the myofibroblasts can have an aerobic glycolytic phenotype, representing a phenotypic change, and would originate from the direct interaction with malignant epithelial cells.

A proteomic analysis was conducted in support of this hypothesis, this mechanism is mediated by the strong downregulation of the expression of caveolin-1 in fibroblasts that are associated with the stroma in breast cancer [142]. The loss and/or downregulation of the expression of caveolin-1 by stromal fibroblasts in breast cancer have been seen as an important biomarker that suggests poor survival [143]. In addition, in these fibroblasts, there is an upregulation of the lactate shuttle expression [144,145].

Other important mechanisms of interaction were identified by Samudio *et al.*, who highlighted the close interaction between mesenchymal stem cells (MSCs) and leukemic cells in the microenvironment of the hematopoietic bone marrow [146]. In particular, the microenvironment is an essential factor, at least for hematopoietic malignancies [147,148], and the exposure of leukemic cells of various lines to the simultaneous co-culture with MSCs under normoxic conditions is associated with mitochondrial membrane potential depolarization, a marked glycolytic switch and increased lactate in the extracellular medium. All of these events appear to be mediated by the expression of the uncoupling protein 2 (UCP2). It is interesting to note that the depolarization of the mitochondrial potential as well as the increased expression of the protein UCP2 are rapid phenomena and that they peak soon after 30 min, and after 120 min, are associated with an increased consumption of O_2_. This phenomenon could lead to an increase in the β-oxidation of fatty acids if we consider that the latter mediates the expression of UCP2 [146,149,150]; however, the same β-oxidation of fatty acids stimulates the expression of uncoupling proteins and in particular of UCP2 [151,152], and the uncoupling and the depolarization of the mitochondrial membrane potential also leads to chemoresistance, a reduction of ROS and the stabilization of the mitochondrial permeability with a consequent reduction in apoptosis [152,153].

Beyond these considerations and the multiple interactions between the microenvironment and cell population, the classic experiments by Mintz and Illmensee underlined the vital importance of the cell population in the development and regulation of tumor growth [154]. In these studies, an embryonic teratoma that originated from a mouse strain expressing a murine dark-haired phenotype and was obtained from six-day-old embryos was grown for 200 generations in female mice with another phenotype (white hair) by peritoneal implantation. For 200 generations, these teratomas were transplanted into a chimeric mouse blastocyst with a white phenotype. The progeny were healthy; some of the progeny expressed an intermediate phenotype and were healthy. Their progeny could bring back the original phenotype (dark hair), were healthy, had a normal structure of the XY chromosomes, produced a healthy seed, and were free of cancer. This result clearly demonstrates the close relationship between the microenvironment and cellular reprogramming. Today, this hypothesis is extremely interesting, especially in the light of CSC theory.

It is also interesting to note a peculiar, seemingly detached aspect of the yeast Saccharomyces cerevisiae. This single-celled organism, which we discussed in the previous section, has the intrinsic pathway of apoptosis. This fact was discovered in the early 90s and was surprising because it was hardly possible to ascribe a finalistic meaning, such as that of programmed cell death, commonly found in metazoans. It has been highlighted by several authors that apoptosis is caused by various environmental conditions, such as nutrient deficiency, and is closely connected to oxidative phosphorylation. The glycolytic phenotype presents a shutdown of the apoptotic process and a clear selective advantage compared to the non-glycolytic phenotype and/or to other non-fermenting microorganisms. This apoptosis block is essential during the early stages of the birth of a colony of Saccharomyces cerevisiae [155]. However, the glycolytic flux, as we have noted, is strongly modular and is used for local reserves. Moreover, this close connection between glycolysis, ATP stores, cell respiration and apoptosis has been clearly highlighted even in eukaryotic cells, both in the normal and in the neoplastic cells [156,157].

## 6. Mitochondria

Mitochondrial homeostasis is essential for life. These organelles probably derive from symbiotic microorganisms and represent a major and crucial step in the transition from anaerobic to aerobic life. These double-membrane organelles, in addition to producing ATP, play a vital role in calcium homeostasis, in the regulation of apoptosis and in the production and disposal of free radicals [158–161]. It is interesting to note that, as previously mentioned in this report, the cellular differentiation of adult stem cells, embryonic stem cells and/or iPSCs is accompanied by a sharp increase in mitochondrial biogenesis, by an increase in oxidative phosphorylation and by a remodeling of the mitochondrial structure [158,162,163]. In addition, the highly hypoxic environment of the uterus and the inherent characteristics of the zygote and then of the blastocyst cause an absence of mitochondria with poorly developed cristae, even one or two per cell, and a strong glycolytic phenotype. During differentiation occurs mitochondrial biogenesis, a very strong acceleration in the rate of synthesis of mDNA. The abolition of a state of oxidative phosphorylation determines the undifferentiation or the maintenance of an embryonic state [158,162–167].

Several mitochondrial alterations are correlated with carcinogenesis and with the transformation and evolution of a neoplasm. We will analyze some examples that have been highlighted in the literature. It is interesting to note that the alterations in the activity of the manganese superoxide dismutase are intrinsically related to carcinogenesis and tumor remission both *in vitro* and *in vivo*. This enzyme, which is encoded by the nucleus and found at the mitochondrial level, is essential for aerobic life and oxygen utilization for energy production. The complete knockout of this gene in mice and Drosophila melanogaster is incompatible with life [168,169] due to the abolition of normal respiratory processes and to the mitochondrial function deficiency. Heterozygous knockout in mice that have only 50% activity with no detected alteration of the immune functions nor accelerated aging but a high increase in the frequency of each type of cancer [170].

We know that the mitochondrial oxygen consumption in cancer cells often does not decrease but increases during the S phase of the cell cycle, even though without any generation of ATP [171]. Numerous tumors are characterized by an overexpression of uncoupling proteins, for instance, in human colorectal cancers [172–174]. In particular, UCP2 appears to be overexpressed in leukemia and in other forms of cancer [146]. In addition, the suppression of its expression by siRNA does not completely abolish the uncoupling but reduces it, suggesting that in addition to this mechanism, there are other mechanisms that lead to an uncoupling of oxidative phosphorylation and to a dissipation of the proton gradient [150]. It is also important to note that pyruvate, of the glutamine metabolism and of the formation of the α-ketoglutarate is essential not only for anaplerotic cycles, *i.e.*, synthesis of pyrimidines and purines, *etc.*, but also for the β-oxidation of fatty acids, as is well expressed in the old biochemical aphorism “fats burn in the fire of carbohydrates” [150,175].

We can also find this particular aspect of the metabolic Warburg effect in stem cells [176]. In fact, in hPSCs, there is O_2_ consumption with futile cycles, whereas the ATP formation occurs through glycolysis. However, we observe not only the decoupling and the presence of uncoupling proteins but also changes in the expression of mitochondrial ATP synthase. The low level of expression of the mitochondrial ATP synthase is associated with a worse prognosis in renal cell carcinoma [177]. In addition, many other functional modifications, polymorphisms and mutations in mDNA and defects in biogenesis and in non-metabolic-related functions have been described in mitochondria [178–180].

In addition to what has been discussed above, we should emphasize that that is an active communication between mitochondria and cell nucleus. In the yeast Saccharomyces cerevisiae, reply of retrograde control (RTG) follows the depolarization of the inner mitochondrial membrane potential, the decrease of ATP stocks or disorders of the electron transport chain [181–184]. This reply is mediated by a sequence of protein complexes (RTG2), which work as sensors of the intracellular ATP level and activate the RTG1/RTG3 complex. This complex leads to, by moving to the nucleus, and by modulating the level of expression of several proteins, a sharp increase in glycolysis and/or oxidative phosphorylation. On the whole, the effect of RTG upregulates glycolysis and a clear increase in the use of glutamine for energy purposes [181,185–187]. Furthermore, RTG2 can also move to the nucleus and regulate chromosomal stability [182].

Ultimately, we must emphasize that carcinogenesis is a process that strongly depends on and is connected to the loss of the normal processes of oxidative phosphorylation, which occurs with cigarette smoking [188,189], in alcoholism [190–192], and in the exposure to several carcinogenic agents, such as 12-hydroxystearic acid and its methyl ester, which demonstrate their carcinogenic action through a decoupling of oxidative phosphorylation [193]. We notice the same phenomenon with many other toxic substances [192]; even several oncogenic viruses cause the loss of oxidative phosphorylation. Viruses that can affect mitochondrial function include the Rous sarcoma virus, Epstein-Barr virus, Kaposi’s sarcoma-associated herpesvirus, human papillomavirus, hepatitis B virus, hepatitis C virus and human T-cell leukemia virus type 1 [194–197].

Otherwise, when the cytoplasm of non-nucleated cells fuses with that cancer cells, even though nuclear alterations remain, the neoplastic state is reprogrammed and abolished [198–200]; healthy organisms can be born even when transplanting cancer cells into blastocysts with healthy mitochondria [201–203].

It is also important to indicate that in the tests of cell fusion and cell hybridization, if the cytoplasm of the healthy non-nucleated cell shows mutations and/or alterations of the mitochondrial functionality, the processes of cancer abolition and reprogramming is suppressed [203,204]. Similarly, we can comment of the classical tests of Mintz’s teratoma that were discussed in the population dynamics section [154].

## 7. Metabolic State, Differentiation and Cell Cycle

As already widely expressed, in the past 50 years, the close reliance on the glycolytic phenotype and on the undifferentiated state have been highlighted. In addition, the glycolytic pathway represents not only a source of ATP but also an efficient anaplerotic system. We observe both a marked glycolytic action and a strong alteration of all of the enzymes that are associated with the tricarboxylic acid cycle and the metabolism of fatty acids and of glutamine. This metabolism is typical of the so-called undifferentiated state.

It is interesting to note that rapidly growing cells such as T lymphocytes, in the course of their clonal expansion, are markedly glycolytic and are characterized by the Warburg effect [205]. Even in the case of normal stimulation with mitotic agents, we observe a shift toward aerobic glycolysis and toward a strong production of lactate, even in rat thymocytes [206], in hepatocytes during liver regeneration [207–209], and wound healing [210,211]. We could likely conclude that an anaerobic metabolism, or rather turning the nutrients into chemical energy in an oxygen-independent manner, is typical of the undifferentiated state and of cycling cells.

The importance of aerobic glycolysis during the rapid cycles of cell division has been documented for many years [212]. During the formation of human trophoblasts and the entire development of homogenesis, changes in the ATP/ADP ratio play essential roles [213]. It was recently shown that during chondrogenesis and during osteoblast and skeletal structure differentiation are synchronous oscillations in the production of ATP and in the ATP/ADP ratio [214]. This synchronization with the other cells derives from the gap junction activity. The blocking of this oscillation abolishes the process of differentiation of the osteoblasts and the formation of the skeletal matrix. In Saccharomyces cerevisiae and in many other cells belonging to both animal and vegetable kingdoms, there are fluctuations in the ATP/ADP ratio.

It is also important to remember that these fluctuations are primarily due to the glycolytic system, which depends on the energy reserves and on the contribution of glucose for its flow; moreover, in yeast and in other cell systems, when reaching a certain population density threshold, a synchronization of the oscillations and a stationary trend occur in unison in all of the cells in a population. This behavior optimizes the energy reserves and represents an important evolution strategy [215–217]. In this way, a particular anaerobic or simply O_2_-independent arrangement cannot be typical of cancer or of other diseases but can be typical of the undifferentiated state and of rapidly growing cells, whereas the cellular differentiation and the improvement of a highly ordered state require efficient oxidative phosphorylation [218]. However, in colon and breast cancers, in melanomas and in many other cancers, there is a direct link between dedifferentiation, advanced disease, and metabolic structure that is typical of the Warburg effect [219–222]. In contrast, in the same cancers, there is a decrease in oxidative phosphorylation, which is fulfilled even for inflammation, carcinogens, polymorphisms in the mtDNA and other conditions, leading to a significant increase in the potential carcinogenicity [223]. Recently, Buravkova *et al.* demonstrated that human mesenchymal cells maintain an undifferentiated state with the lowering of the ATP stores [224]; *i.e.*, low ATP levels are enough to maintain a low degree of differentiation and an undifferentiated state. It is interesting to highlight how even one of the most famous differentiating agents, retinoic acid and retinoids in general, mediate their effects through a remodeling of the metabolism and a clear increase in oxidative phosphorylation. Xun *et al.* and Saumet *et al.* recently stressed again the close connection between differentiation and cell respiration [225,226].

## 8. Notes on Thermodynamics

The glycolysis or Embden-Meyerhof-Parnas pathway is one of the most preserved and phylogenetically old metabolic pathways that produce energy in the form of ATP.

It is common knowledge that glycolysis, which produces phosphoanhydrides by phosphorylation in the substratum, is less streamlined than is oxidative phosphorylation; one mole of glucose through the glycolytic pathway will produce only 2 moles of ATP compared to the 32 moles that are derived by oxidative phosphorylation.

Nevertheless, more than the efficiency of the ATP molecules (moles of ATP per mole of substrate), the synthesis speed or synthesis rate of ATP per unit of time (moles of ATP per unit time) is also important. The phosphorylation processes in the substratum, although less efficient, are faster.

The trade-off between a low efficiency mechanism with a fast rate and a high efficiency mechanism with a low speed of production per unit of time is a discriminating evolutionary factor and is very important in cellular physiology [98,227].

Unicellular facultative anaerobic organisms take advantage of colonizing other cellular colonies if they use ATP production at a fast rate but low efficiency. Specifically, the choice between efficiency and rate or a better balance between the yield and rate is extremely important during the first phases of the colonization of a field or the invasion of another colony. In terms of evolution, the competition for resources and for a new colony genesis prefers fermentation for the unicellular organisms, as has been demonstrated for many anaerobic facultative microorganisms, such as yeasts, which use fermentation even in the presence of O_2_ [228–230]. It is interesting to observe the behavior of molds, such as the Mucor racemosus species, which uses fermentation in a unicellular state; during the passage to multicellular forms, as in during mycelium formation, the development and the passage to oxidative phosphorylation originate. In fact, the inhibition or knockout of oxidative phosphorylation prevents the passage from the unicellular form to the multicellular one (mycelium). Pfeiffer *et al.* demonstrated that in the competition between unicellular organisms of different colonies and in the passage to metazoan organisms, we can speak about the “tragedy of commons” that is largely applied in economics and cooperativism for metazoan organisms, which pass to oxidative phosphorylation [98]. In this meaning, the fermentation in the cancerous cell is the passage to a non-cooperative form.

The study Pfeiffer *et al.* includes an exact mathematic treatment. As is known from the second law of thermodynamics, the free energy or rather the actually useful energy to perform work, is given by the following relationship, dG = dH − TdS. Because the vital reactions are isothermal, we approve the temperature in the differential dG = dH − dTS. In the case of ATP hydrolysis, as widely known, the following relationship applies,

(1) $$\Delta \text{G}=\Delta {\text{G}}^{\circ }+\text{RT ln}\frac{\left[\text{ADP}][\text{Pi}\right]}{\left[\text{ATP}\right]}$$

whereas the relationship linking the equilibrium constant and the ΔG is expressed by the following exponential relationship,

(2) $$\text{Keq}={\text{e}}^{-\Delta \text{G}/\text{RT}}$$

which is actually an assumption and a simplification as we should account for the pH conditions, complexometric equilibrium with Mg^2+^, activities of all of the species in solution and several other variables.

However, it is evident that there is a close connection between the concentration of ATP and the free energy of hydrolysis, as is well known from the most essential rudiments of thermodynamics. In biochemistry, cells are structures that are far from thermodynamic and chemical equilibria; indeed the reaching of a ΔG = 0, Keq = 1 or rather of the steady state or of equilibrium coincides with death and the end of all vital activities. Moreover, even minimal changes in the ΔG of ATP hydrolysis are extremely dangerous and sharply affect cell homeostasis.

Although it is intuitive to think that a lowering of the energy or a lowering of the ATP/ADP ratio is dangerous for cellular life, an increase is also dangerous because it causes the loss of ionic homeostasis and deep changes in the Gibbs-Donnan balance. In fact, there is a strict relationship between ionic homeostasis and ΔG_ATP_ [231].

The importance of free energy from ATP hydrolysis and of its variations appears to be capital in every aspect of cell life but even more so for the maintenance of ionic homeostasis and of the concentration gradients that represent the essential basis of all of the electrophysiological phenomena.

As emphasized by Veech *et al.* in a review entitled “The resting membrane potential of cells are measures of electrical work, not of ionic currents” [232], we can state that living systems create membrane potentials with an electrostatic force,

(3) $$\text{F}=\frac{\text{Q}1\text{Q}2}{4\pi {ɛ}_{0}{ɛ}_{\text{r}}{\text{r}}^{2}}$$

producing the potential,

(4) $$\text{E}=\frac{\text{Q}}{4\pi {ɛ}_{0}{ɛ}_{\text{r}}\text{r}}$$

In turn, the balance potential is given through the balance that is reached between two opposing forces that fulfill the osmotic and electrical work, or rather Wc = We with $\text{Wc}=\Delta \text{G}\;\text{concentration energy}=\text{RT ln}\frac{\left[\text{Ion }{\text{x}}^{\text{z}}\right]}{\left[\text{Ion }{\text{x}}^{\text{z}}\right]}$ and We = ΔG electrochemical potential = zFE[IonN^Z^].

As between these two phases, there is a potential in which WC = −We. In absolute value we get a connection which, even if rounding up and down, is well described through Nernst’s equation,

(5) $$\text{E}{\left[{\text{IonN}}^{\text{Z}}\right]}^{\text{out}/\text{in}}=\frac{\text{RT}}{\text{zF}}\text{ln}\frac{[{\text{IonN}}^{\text{Z}}]\text{out}}{[{\text{IonN}}^{\text{Z}}]\text{in}}$$

and is well summarized with Gibbs-Donnan’s balance. In addition, Veech *et al.* believe that the membrane potential, through biological membranes, should not be ascribed to the diffusion potential nor to a kinetic analysis of this issue as gracefully illustrated by Goldman in 1943 by adopting the theory of constant electric field; moreover by completing Nernst-Plank’s equation, the origin of the membrane potential should be ascribed to the electrochemical work on the basis of the following relation, $\frac{\text{RTln}{\left[\text{Permeant }{\text{Ion}}^{\text{z}}\right]}^{\text{out}/\text{in}}}{\text{zF}}$.

This idea implies a close connection between the electrochemical work and the ΔG of ATP hydrolysis, which supplies the essential driving force through the action of membrane 3Na^+^/2K^+^-ATPase, which is essential and centrally connected among all of the electrophysiological phenomena and the potential of cell phosphorylation.

To better understand some ideas, we can imagine an ideal cell. This cell has to constantly fulfill a specific osmotic function to maintain its integrity. To be more precise, we can imagine the work that the membrane 3Na^+^/2K^+^-ATPase has performed. Masuda *et al.*, in studying the electrophysiology of cardiomyocytes, demonstrated that the relationship among the 3Na^+^/2K^+^-ATPase, the membrane potential and the ATP hydrolysis is supported by the following equation [233]; otherwise, the close connection between the law of mass action and the dG of ATP hydrolysis is highlighted by considering that by merely varying the [Pi] we experimentally obtain some predictable variations by means of the following generalization,

(6) $$\Delta {\text{G}}^{\circ }\text{ATPHydr}-\text{RT ln}\frac{\Sigma [\text{ATP}]}{\left[\Sigma \text{ADP}][\Sigma \text{Pi}\right]}+\text{RT ln}\frac{{\left[{\text{Na}}^{+}\right]}_{\text{o}}^{3}\;{\left[{\text{K}}^{+}\right]}_{\text{i}}^{3}}{{\left[{\text{Na}}^{+}\right]}_{\text{i}}^{3}{\left[{\text{K}}^{+}\right]}_{\text{o}}^{3}}=0$$

we can understand how the close relationship between ATP and its production and how diffusion is basic in all of the ionic processes and must be considered not as an inactive event but as an active expression of electrochemical work. Therefore, if in the neoplastic cell there is a production of energy that varies and is not constant in time, we can understand the reason for the numerous electrophysiological alterations in neoplastic cells, or rather even of the alterations in the activity of 3Na^+^/2K^+^-ATPase [234,235].

However, if we can affirm that the major part of energy, ATP in tumoral cells, is derived from glycolysis, we must consider the prevalent oscillating feature of this biochemical pathway. This event has already been demonstrated [215–217,236]. This oscillatory behavior is produced by many causes and carries within it numerous observations, Yang *et al.* has shown that in cardiomyocytes, conditions of anoxia or of oxidative phosphorylation block produce an oscillatory behavior of glycolysis and a marked imbalance between supply and demand in energy terms [217].

These data have recently been confirmed by Ganitkevich *et al.* [237] as well as in tumor cells, which produce most of their ATP through glycolysis [238,239]. Many studies have demonstrated the prevalent feature of PFK-1 in maintaining and regulating glycolysis. It is peculiar that this enzyme is stimulated by its own product (ADP) and in its turn it is inhibited by the final product of the glycolytic pathway (ATP). This positive/negative feedback is very important in establishing and regulating the oscillatory phases [240]. Many studies have demonstrated that, in such a system, the ATP production and the ATP/ADP ratio are exposed to sudden oscillations according to the cycles. However, these oscillations are responsible and synchronous with the oscillations of transmembrane Ca^2+^ and K^+^. In addition, the same glycolytic system provides an increase of [Na^+^] through both of glucose/Na^+^ cotransport and through H^+^/Na^+^ symport, increasing the activity of the pump 3Na^+^/2K^+^-ATPase and an ulterior increase of [ADP]i, which could stimulate the PFK-1 [241,242].

Many studies have demonstrated a pronounced alteration of the ATP/ADP ratio in neoplastic cells with sudden changes and a prevalence toward low [ATP]i levels. Several works of magnetic resonance spectroscopy (31P–NMR) have emphasized that in isolated cells, *in vivo* during carcinogenesis and in both animal and human patterns, are intense variations at the levels of ATP (free ATP and complexed Mg ATP), ADP and Pi. In particular, Baluch *et al.* have underlined a marked and substantial decrease in the ATP levels, ATP/ADP ratio, free Mg^2+^ and free energy of ATP hydrolysis in breast cancer [243]; in addition, similar elements have already been highlighted in Ehrlich ascites tumor cells by Navon *et al.* [244] and even by Ross *et al.* in the *in vivo* murine glioma in intracerebral xero-transplants [245]. Furthermore, similar events occur in mammary neoplasias both *in vitro* and *in vivo*, which have been studied using the same technique [246–249]. Recently, Golinska *et al.* demonstrated that the cancer cells of hepatoma and of other hepatocellular carcinomas, even in the presence of a marked decrease in the inducible factor from hypoxia (HIF-1), independently of the modulation of gene expression and of mediated effects through proteomic mechanisms, significantly decrease the ATP levels and increase those of AMP [250]; these authors also present the stable retention of a stationary glycolytic state that is maintained through the allosteric effect of AMP on phosphofructokinase-1. This behavior is also found in undifferentiated cells belonging to the stem cells that govern self renewal (adult stem cells, ASC) [51,162,251,252].

In fact, many other independent studies using various techniques have demonstrated that neoplastic and stem cells have less stock of ATP, producing less susceptibility to apoptosis, to carcinogenesis and an inherent instability of all of the cellular homeostatic states [176,253,254]. It is clear from the references that, in undifferentiated cells, in neoplastic cells and in all of the system that are prevalently or exclusively glycolytic, there are sudden changes in the ΔG_ATP_ and on the whole in the capacity to do work or in other words “the differentiation work”.

As stated by Frieden and Gatenby, living systems, like everything else in the universe, submit to the laws of thermodynamics and completely satisfy the 2nd principle [255]; however, these systems present some peculiarities compared to non-living systems:

(a) Living systems show periodic structures with low (but not minimal) levels of entropy; these structures alternate with those with a disordered degree (greater randomness), such as the plasma membrane and chromosomes alternating with the cytoplasm;
(b) Living systems use power sources from chemical reactions that far from the thermodynamic equilibrium to maintain order and ATP hydrolysis; even though there are systems with highly ordered structures (with low entropy), these systems also have low energy (in balance), such as crystals;
(c) Living systems can replicate, and the cell split also represents a means of entropy decrease, thus a typical cell, as it finishes its cell loop, increases its order degree; through the split cells with a greater degree of randomness; the living systems then die or reach thermodynamic equilibrium (ΔG = 0);
(d) In conclusion, living systems see their entropy and energy varying through time by maintaining certain parameters; the randomness is neither too high nor too low and oscillating between the maximum and minimum values occurs.

From these observations, these authors have concluded the following general postulates:

Living systems are non-equilibrium opened but locally delimited, thermodynamic systems that use information to convert environmental energy into order. The survival of a living structure requires a stable state of order despite continuous thermal and mechanical perturbations.

The stability of a living system requires its information content to be maintained at an extreme.

To explain and rationalize this complexity, Frieden and Gatenby have emphasized the importance of information codes or rather the importance of exact information, for example the genetic code, the histone code (epigenetics) and so on [255].

Therefore, the codes of information, such as “theory of nets”, produce a state of low or high energy. Recently, Teschendorff and Severini, using methods of mathematical inference and the analysis of gene expression patterns, expressed a method to validate the cellular network entropy [256]. These authors adopted a systems view and considered the sample’s network entropy, a measure of the signaling pathway promiscuity, which is computable from a sample’s genome-wide expression profile [257,258]. These authors also indicate the high level of causality in the pattern of gene expression and in the cellular network entropy, both in cancer and with a progression and increase in the metastatic cells. Lastly, these authors demonstrate a clear decreasing gradient of entropy during cellular differentiation, from undifferentiated cells (hESC, iPSCs, rSC, *etc.*) to differentiated cells, similar to the decreasing cellular network entropy during the differentiation of leukemic HL-60 after all-trans retinoic acid treatment [258]. The change in the cellular network entropy guides cellular differentiation through a classic Waddington’s differentiation landscape [259].

Referencing a brief mention of the theory of ontogeny and of the development of the Prigogine-Wiame theory, during development, growth and aging, there is a constant decrease in the dissipation function values, as follows:

(7) $$\frac{\text{d}\Psi }{\text{dt}}\leq 0$$

according to this theory, Ψ = qO_2_ + q_Glyc_, in other words, it is equal to the sum of the intensity of the respiration (consumption of O_2_) and the glycolysis, the only thermodynamic variables in play:

(8) $$\Psi =\frac{T}{V}\frac{\text{dS}}{\text{dt}}=\sum_{\text{j}=1}^{\text{n}}J\;\text{i }X\;\text{i }(\text{steady state})$$

where Ψ is the dissipation function, *T* is the absolute temperature, *V* is the volume or mass of the system, $\frac{\text{dS}}{\text{dt}}$ is the entropy production, *J* is the specific thermodynamic flows, and *X* is the thermodynamic forces.

That is, the life and the persistence in life of an individual require the settling of a stationary state and of a precise trajectory in which no entropy is created. In this sense, a living system represents a dissipative structure.

According to the theory of ontogeny and the development of the Prigogine-Wiame theory, the development of an individual may be associated with a transient increase in the values of the function Ψ with marked deviation from the trajectory and from the stationary state, at low energy and low entropy at the expense of the environment. This deviation occurs at the expense of the environment and especially in the fertilized oocyte in which occurs a transitional state of higher randomness that corresponds to an increase in the function of dispersion [260] in such a way that it realizes albeit transiently a condition in which:

(9) $$\frac{d\Psi m}{dt}>0$$

(10) $$\Psi m=\frac{T}{V}\frac{dSm}{dt}=\sum_{k=i}^{n}J\;k\;X\;k\leq 0$$

this theory is formulated for thermodynamic systems that are not in equilibrium but are close to balance; in other words, this theory describes a trend that is valid for linear and irreversible processes in the proximity of the thermodynamic equilibrium. Therefore, this theory has been the subject of criticism and concern [261,262].

If we consider a true biological situation, according to what Prigogine-Wiame expressed and to the evidence, we can conclude that the actual situation of the differentiation of a cell is really opposite and symmetrical of what the Prigogine-Wiame theory said, where the ideal development of a cell during the differentiation process matches a decrease in the structural randomness [258] and an increase in the specific rate of production of entropy in the sense of an increase of the mitochondrial activity until it reaches a perfect stationary state. While the real situation at the fecundated oocyte level seems to be more complex and heterogeneous than this theory states, in fact, an oxidative burst and a dissipation of its energy from the decoupling of oxidative phosphorylation have not been observed [260,263,264].

Regardless of this aspect, it is clear that the capacity of doing “differentiation work” is expressly given by oxidative phosphorylation and finally by ATP availability and by its consumption per unit of time. It is worth noting that there is often confusion between O_2_ consumption and oxidative phosphorylation. Often in neoplastic cells and in undifferentiated cells, this phosphorylation can also occur at a high level but is decoupled from phosphorylation so that oxidative phosphorylation is substituted with substrate-level phosphorylation. However, decoupled respiration could play a cytoprotective role for O_2_ toxicity [265].

## 9. Conclusions

We have known for more than 150 years that the neoplastic cells are associated with an undifferentiated marked state, and the great German pathologist Julius Friedrich Cohnheim, the more famous assistant of Rudolf Virchow, stated that the origin of cancer could be traced to the presence of embryonic tissue residues [266]. The recent knowledge of the origin of cancer and of the CSCs confirms the pivotal role of an undifferentiated population that is responsible for the maintenance and renewal of neoplasms.

It is also important to note that each neoplasm is not the result of a specific monoclonal population but of a heterogeneous population of cells, which has its own stem compartment and neoplastic cells in a progressive, albeit aberrant, degree of differentiation.

As we have seen in the course of this study, a predominantly glycolytic phenotype, with low levels of ATP, of ROS, and of oxidative phosphorylation, characterized by the presence of anaplerotic cycles, is typical of the undifferentiated state and of a high number cell division cycles in invertebrates and in vertebrates. Conversely, during cellular differentiation, we observe increased levels of ATP and ROS production and mitochondrial biogenesis, increased oxidative phosphorylation, and decreased decoupling of oxidative phosphorylation.

Although in the last 60 years cell biology has emphasized the genetic/epigenetic aspects that are linked to carcinogenesis and cancer, it has been clearly demonstrated in eukaryotes, both in simple organisms such as Saccharomyces cerevisiae, in other single-celled organisms and in metazoans, that the set of all of the metabolic reactions (metabolome) can significantly and strongly affect the level of expression of numerous genes through epigenetic mechanisms, such as the modulation of histone acetylation, cytosine methylation and also demethylase and deacetylase.

In addition, the ATP/ADP ratio has been valued as an essential factor in the regulation of the cell cycle, both during S phase, G0/G1 phase and all cellular life. Cellular differentiation and the maintenance of the differentiated state require considerable work and are necessarily coupled to special mechanisms that are necessary to produce the correct amount of energy without which neither cellular differentiation nor the maintenance of a highly ordered state, such as that of a differentiated cell, can occur. In summary, the careful examination of these data and of the literature shows that cellular differentiation requires an efficient mechanism of oxidative phosphorylation and constant metabolic cycles. However, if we consider the neoplastic problem that is sustained by a stem cell compartment and by a lack of cellular differentiation, then the Warburg effect is not to be considered as the special chief characteristic of cancer cells but as the typical aspect of the undifferentiated state, *i.e.*, an intrinsic characteristic of the low cellular differentiation. In addition, this effect reaches one of the highest chaotic expression levels in neoplasms.

Analyzing a graph of the logarithm of global cancer incidence *vs.* age, we note that this graph is similar to a straight line with a gradient of 6–7. According to the value of the power-law exponent, we can conclude that the genesis of a neoplasm derives from 6–7 independent events [267,268]. Consequently, the lack of development in the oxidative phosphorylation mechanism and the permanent shift towards a glycolytic phenotype could be seen as a necessary but not sufficient causal factors for neoplasms where we observe the loss of one of the fundamental requirements for cellular differentiation.

Therefore, neoplastic disease could be identified as being characterized by a lack of differentiation, and its driving force could be traced in the changes of the metabolome.

**Figure 2.**
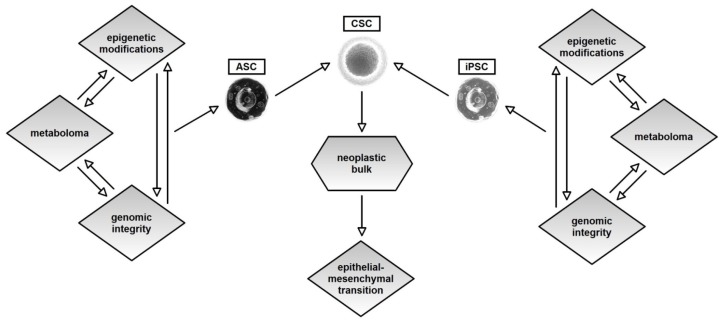
Figure 2 briefly summarizes our model in which, at the origin of a neoplasia, there are not only changes, genetic or epigenetic, but also triple synchronous changes in three systems, metabolic, genetic and epigenetic. We propose an integrative model that is based on cooperative and multiple changes in these three systems. Very probably, a change in one of these cellular systems is not sufficient cause but serves to prepare the cell. From all of these facts and from the growing evidence supporting the CSC theory, we can hypothesize that the transformation process, which foresees this triple progressive change in the three basis systems, occurs in stem cells that preside over self renewal (ASC) or in somatic cells that, by the lost of the correct metabolome and by the change in numerous genetic and epigenetic systems, proceed to a process of somatic rescheduling (iPSC). Certainly, the energetic homeostasis, mitochondrial function and the metabolome as a whole play roles of equal importance in genetic or epigenetic phenomena. From this perspective, more care and attention should be given to other well-know factors.

In the end, Figure 2 summarizes our model, in which a neoplasm arises from a single cell stem (ASC) or from an iPSC, in accordance with the current facts about the stem origin of cancer.

The specific metabolic phenotype known here as the Warburg effect is not considered as a metabolic signature that is acquired during the oncogenesis process but represents an aberrant expression of a metabolic layout that is typical of the undifferentiated state. The permanent shift toward this specific metabolic signature is not shown here as the only cause of cancer (Warburg hypothesis) but as an essential contributory cause. Where neoplasms can be placed even as a pathology of aberrant differentiation, their exact development and the maintenance of a differentiated cell state (with less randomness) are due to thermochemical functions. Therefore, we state that as a contributory cause of the neoplastic development that is related to undifferentiated cells that there is a gradual and irreversible establishment of an undifferentiated state, with a gradual or complete loss of oxidative phosphorylation, rather than respiration in itself, which, as we have noted, is often present in neoplasms.

This fact leads to a deep alteration of numerous epigenetic mechanisms, greater gene instability, greater structural randomness, a marked resistance to apoptosis and many other alterations that are intrinsic to neoplasms.

It is likely that one of the most important challenges of cancer research is determining the biochemical, chemical-physical and biomolecular mechanisms that drive the failure of the correct development of oxidative phosphorylation mechanisms in the original stem precursor (ASC) or iPSC [269,270].

Lastly, the energetic and metabolic state, based on our way of thinking, could represent the central link between genetic/epigenetic instability and the CSC theory considering that a characteristic and essential feature of each neoplasm is the lack of differentiation [188,193,271–284].

## References

[1] Jacob F., Morton K. (1937). The transmission of leukemia of mice with a single cell. Am. J. Cancer.

[2] Kruse J.P., Gu W. (2009). Modes of p53 regulation. Cell.

[3] Olovnikov I.A., Kravchenko J.E., Chumakov P.M. (2009). Homeostatic functions of the p53 tumor suppressor: Regulation of energy metabolism and antioxidant defense. Semin. Cancer Biol.

[4] Sonnenschein C., Soto A.M. (2000). Somatic mutation theory of carcinogenesis: Why it should be dropped and replaced. Mol. Carcinog.

[5] Loeb L.A. (2001). A mutator phenotype in Cancer. Cancer Res.

[6] Nowell P.C. (2002). Tumor progression: A brief historical perspective. Semin. Cancer Biol.

[7] Baker S.G., Kramer B.S. (2007). Paradoxes in carcinogenesis: New opportunities for research directions. BMC Cancer.

[8] Mori H., Colman S.M., Xiao Z., Ford A.M., Healy L.E., Donaldson C., Hows J.M., Navarrete C., Greaves M. (2002). Chromosome translocations and covert leukemic clones are generated during normal fetal development. Proc. Natl. Acad. Sci. USA.

[9] Sengupta A., Cancelas J.A. (2010). Cancer stem cells: A stride towards cancer cure?. J. Cell Physiol.

[10] Lapidot T., Sirard C., Vormoor J., Murdoch B., Hoang T., Caceres-Cortes J., Minden M., Paterson B., Caligiuri M.A., Dick J.E. (1994). A cell initiating human acute myeloid leukaemia after transplantation into SCID mice. Nature.

[11] Bonnet D., Dick J.E. (1997). Human acute myeloid leukemia is organized as a hierarchy that originates from a primitive hematopoietic. Cell. Nat. Med.

[12] Al-Hajj M. (2007). Cancer stem cells and oncology therapeutics. Curr. Opin. Oncol.

[13] Singh S.K., Clarke I.D., Terasaki M., Bonn V.E., Hawkins C., Squire J., Dirks P.B. (2003). Identification of a cancer stem cell in human brain tumors. Cancer Res.

[14] He J., Liu Y., Lubman D.M. (2012). Targeting glioblastoma stem cells: Cell surface markers. Curr. Med. Chem.

[15] O’Flaherty J.D., Barr M., Fennell D., Richard D., Reynolds J., O’Leary J., O’Byrne K. (2012). The cancer stem-cell hypothesis: Its emerging role in lung cancer biology and its relevance for future therapy. J. Thorac. Oncol.

[16] Dhawan P., Ahmad R., Srivastava A.S., Singh A.B. (2011). Cancer stem cells and colorectal cancer: An overview. Curr. Top. Med. Chem.

[17] Zhang S., Balch C., Chan M.W., Lai H.C., Matei D., Schilder J.M., Yan P.S., Huang T.H., Nephew K.P. (2008). Identification and characterization of ovarian cancer-initiating cells from primary human tumors. Cancer Res.

[18] Ahmed N., Abubaker K., Findlay J.K. (2013). Ovarian cancer stem cells: Molecular concepts and relevance as therapeutic targets. Mol. Aspects Med.

[19] Gou S., Liu T., Wang C., Yin T., Li K., Yang M., Zhou J. (2007). Establishment of clonal colony-forming assay for propagation of pancreatic cancer cells with stem cell properties. Pancreas.

[20] Hamada S., Masamune A., Takikawa T., Suzuki N., Kikuta K., Hirota M., Hamada H., Kobune M., Satoh K., Shimosegawa T. (2012). Pancreatic stellate cells enhance stem cell-like phenotypes in pancreatic cancer cells. Biochem. Biophys. Res. Commun.

[21] Lang S.H., Frame F.M., Collins A.T. (2009). Prostate cancer stem cells. J. Pathol.

[22] Malaguarnera R., Frasca F., Garozzo A., Gianì F., Pandini G., Vella V., Vigneri R., Belfiore A. (2010). Insulin receptor isoforms and insulin-like growth factor receptor in human follicular cell precursors from papillary thyroid cancer and normal thyroid. J. Clin. Endocrinol. Metab.

[23] Lorico A., Rappa G. (2011). Phenotypic heterogeneity of breast cancer stem cells. J. Oncol.

[24] Curtin J.C., Lorenzi M.V. (2010). Drug discovery approaches to target Wnt signaling in cancer stem cells. Oncotarget.

[25] Xia T., Jiang H., Li C., Tian M., Zhang H. (2012). Molecular imaging in tracking tumor stem-like cells. J. Biomed. Biotechnol.

[26] Cukierman E., Bassi D.E. (2012). The mesenchymal tumor microenvironment: A drug-resistant niche. Cell Adh. Migr.

[27] Sell S. (2010). On the stem cell origin of Cancer. Am. J. Pathol.

[28] Reya T., Morrison S.J., Clarke M.F., Weissman I.L. (2001). Stem cells, cancer, and cancer stem cells. Nature.

[29] Peitzsch C., Kurth I., Kunz-Schughart L., Baumann M., Dubrovska A. (2013). Discovery of the cancer stem cell related determinants of radioresistance. Radiother. Oncol.

[30] Wicha M.S., Liu S., Dontu G. (2006). Cancer stem cells: An old idea—A paradigm shift. Cancer Res.

[31] Siggins R.W., Zhang P., Welsh D., Lecapitaine N.J., Nelson S. (2008). Stem cells, phenotypic inversion, and differentiation. Int. J. Clin. Exp. Med.

[32] Kondoh H., Lleonart M.E., Bernard D., Gil J. (2007). Protection from oxidative stress by enhanced glycolysis; a possible mechanism of cellular immortalization. Histol. Histopathol.

[33] Kondoh H., Lleonart M.E., Nakashima Y., Yokode M., Tanaka M., Bernard D., Gil J., Beach D. (2007). A high glycolytic flux supports the proliferative potential of murine embryonic stem cells. Antioxid. Redox Signal.

[34] Kraft C.S., LeMoine C.M., Lyons C.N., Michaud D., Mueller C.R., Moyes C.D. (2006). Control of mitochondrial biogenesis during myogenesis. Am. J. Physiol. Cell Physiol.

[35] Chen C.T., Shih Y.R., Kuo T.K., Lee O.K., Wei Y.H. (2008). Coordinated changes of mitochondrial biogenesis and antioxidant enzymes during osteogenic differentiation of human mesenchymal stem cells. Stem Cells.

[36] Stringari C., Edwards R.A., Pate K.T., Waterman M.L., Donovan P.J., Gratton E. (2012). Metabolic trajectory of cellular differentiation in small intestine by phasor fluorescence lifetime microscopy of NADH. Sci. Rep.

[37] Wright B.K., Andrews L.M., Markham J., Jones M.R., Stringari C., Digman M.A., Gratton E. (2012). NADH distribution in live progenitor stem cells by phasor-fluorescence lifetime image microscopy. Biophys. J.

[38] Sen A., Damm V.T., Cox R.T. (2013). Drosophila clueless is highly expressed in larval neuroblasts, affects mitochondrial localization and suppresses mitochondrial oxidative damage. PLoS One.

[39] Legname A.H., Salomón de Legname H. (1980). Changes in the oxidative metabolism during maturation of amphibian oocytes. J. Embryol. Exp. Morphol.

[40] Wales R.G. (1986). Measurement of metabolic turnover in single mouse embryos. J. Reprod. Fertil.

[41] Takahashi K., Yamanaka S. (2006). Induction of pluripotent stem cells from mouse embryonic and adult fibroblast cultures by defined factors. Cell.

[42] Nelson T.J., Terzic A. (2011). Induced pluripotent stem cells: An emerging theranostics platform. Clin. Pharmacol. Ther.

[43] Prigione A., Adjaye J. (2010). Modulation of mitochondrial biogenesis and bioenergetic metabolism upon *in vitro* and *in vivo* differentiation of human ES and iPS cells. Int. J. Dev. Biol.

[44] Vazquez-Martin A., Corominas-Faja B., Cufi S., Vellon L., Oliveras-Ferraros C., Menendez O.J., Joven J., Lupu R., Menendez J.A. (2013). The mitochondrial H^+^-ATP synthase and the lipogenic switch: New core components of metabolic reprogramming in induced pluripotent stem (iPS) cells. Cell Cycle.

[45] Folmes C.D., Nelson T.J., Terzic A. (2011). Energy metabolism in nuclear reprogramming. Biomark. Med.

[46] Panopoulos A.D., Yanes O., Ruiz S., Kida Y.S., Diep D., Tautenhahn R., Herrerías A., Batchelder E.M., Plongthongkum N., Lutz M. (2012). The metabolome of induced pluripotent stem cells reveals metabolic changes occurring in somatic cell reprogramming. Cell Res.

[47] Westermann B. (2010). Mitochondrial fusion and fission in cell life and death. Nat. Rev. Mol. Cell Biol.

[48] Varum S., Momcilović O., Castro C., Ben-Yehudah A., Ramalho-Santos J., Navara C.S. (2009). Enhancement of human embryonic stem cell pluripotency through inhibition of the mitochondrial respiratory chain. Stem Cell Res.

[49] Folmes C.D., Nelson T.J., Martinez-Fernandez A., Arrell D.K., Lindor J.Z., Dzeja P.P., Ikeda Y., Perez-Terzic C., Terzic A. (2011). Somatic oxidative bioenergetics transitions into pluripotency—dependent glycolysis to facilitate nuclear reprogramming. Cell Metab.

[50] Ding D.F., Li X.F., Xu H., Wang Z., Liang Q.Q., Li C.G., Wang Y.J. (2013). Mechanism of resveratrol on the promotion of induced pluripotent stem cells. J. Integr. Med.

[51] Varum S., Rodrigues A.S., Moura M.B., Momcilovic O., Easley C.A., Ramalho-Santos J., van Houten B., Schatten G. (2011). Energy metabolism in human pluripotent stem cells and their differentiated counterparts. PLoS One.

[52] Abu Dawud R., Schreiber K., Schomburg D., Adjaye J. (2012). Human embryonic stem cells and embryonal carcinoma cells have overlapping and distinct metabolic signatures. PLoS One.

[53] Palorini R., Votta G., Balestrieri C., Monestiroli A., Olivieri S., Vento R., Chiaradonna F. (2014). Energy metabolism characterization of a novel cancer stem cell-like line 3AB-OS. J. Cell Biochem.

[54] Huber M.A., Kraut N., Beug H. (2005). Molecular requirements for epithelial-mesenchymal transition during tumor progression. Curr. Opin. Cell Biol.

[55] Tarin D., Thompson E.W., Newgreen D.F. (2005). The fallacy of epithelial mesenchymal transition in neoplasia. Cancer Res.

[56] Smith D.G., Sturmey R.G. (2013). Parallels between embryo and cancer cell metabolism. Biochem. Soc. Trans.

[57] Warburg O., Wind F., Negelein E. (1927). The metabolism of tumors in the body. J. Gen. Physiol.

[58] Tennant D.A., Durán R.V., Gottlieb E. (2010). Targeting metabolic transformation for cancer therapy. Nat. Rev. Cancer.

[59] Warburg O. (1956). On respiratory impairment in cancer cells. Science.

[60] Seyfried T.N., Shelton L.M. (2010). Cancer as a metabolic disease. Nutr. Metab.

[61] Vander Heiden M.G., Cantley L.C., Thompson C.B. (2009). Understanding the Warburg effect: The metabolic requirements of cell proliferation. Science.

[62] Gatenby R.A., Gillies R.J. (2004). Why do cancers have high aerobic glycolysis?. Nat. Rev. Cancer.

[63] Lynen F. (1951). Die rolle der phosphorsaeure bei dehydrierungsvorgaengen und ihre biologische bedeutung. Naturwissenschaften.

[64] Kim J.W., Dang C.V. (2006). Cancer’s molecular sweet tooth and the Warburg effect. Cancer Res.

[65] Fantin V.R., St-Pierre J., Leder P. (2006). Attenuation of LDH—A expression uncovers a link between glycolysis, mitochondrial physiology, and tumor maintenance. Cancer Cell.

[66] Patel M.S., Korotchkina L.G. (2001). Regulation of mammalian pyruvate dehydrogenase complex by phosphorylation: Complexity of multiple phosphorylation sites and kinases. Exp. Mol. Med.

[67] Goetze K., Walenta S., Ksiazkiewicz M., Kunz-Schughart L.A., Mueller-Klieser W. (2011). Lactate enhances motility of tumor cells and inhibits monocyte migration and cytokine release. Int. J. Oncol.

[68] Hirschhaeuser F., Sattler U.G., Mueller-Klieser W. (2011). Lactate: A metabolic key player in cancer. Cancer Res.

[69] Végran F., Boidot R., Michiels C., Sonveaux P., Feron O. (2011). Lactate influx through the endothelial cell monocarboxylate transporter MCT1 supports an NF-κB/IL-8 pathway that drives tumor angiogenesis. Cancer Res.

[70] Kennedy K.M., Dewhirst M.W. (2010). Tumor metabolism of lactate: The influence and therapeutic potential for MCT and CD147 regulation. Futur. Oncol.

[71] Walenta S., Mueller-Klieser W.F. (2004). Lactate: Mirror and motor of tumor malignancy. Semin. Radiat. Oncol.

[72] Eigentler T.K., Figl A., Krex D., Mohr P., Mauch C., Rass K., Bostroem A., Heese O., Koelbl O., Garbe C. (2011). Number of metastases, serum lactate dehydrogenase level, and type of treatment are prognostic factors in patients with brain metastases of malignant melanoma. Cancer.

[73] Koukourakis M.I., Giatromanolaki A., Sivridis E., Gatter K.C., Trarbach T., Folprecht G., Shi M.M., Lebwohl D., Jalava T., Laurent D. (2011). Prognostic and predictive role of lactate dehydrogenase 5 expression in colorectal cancer patients treated with PTK787/ZK 222584 (vatalanib) antiangiogenic therapy. Clin. Cancer Res.

[74] Li G., Gao J., Tao Y.L., Xu B.Q., Tu Z.W., Liu Z.G., Zeng M.S., Xia Y.F. (2012). Increased pretreatment levels of serum LDH and ALP as poor prognostic factors for nasopharyngeal carcinoma. Chin. J. Cancer.

[75] Xie H., Valera V.A., Merino M.J., Amato A.M., Signoretti S., Linehan W.M., Sukhatme V.P., Seth P. (2009). LDH–A inhibition, a therapeutic strategy for treatment of hereditary leiomyomatosis and renal cell Cancer. Mol. Cancer Ther.

[76] Szanto I., Rubbia-Brandt L., Kiss P., Steger K., Banfi B., Kovari E., Herrmann F., Hadengue A., Krause K.H. (2005). Expression of NOX1, a superoxide-generating NADPH oxidase, in colon cancer and inflammatory bowel disease. J. Pathol.

[77] Kawahara T., Lambeth J.D. (2007). Molecular evolution of Phox-related regulatory subunits for NADPH oxidase enzymes. BMC Evol. Biol.

[78] Lu W., Hu Y., Chen G., Chen Z., Zhang H., Wang F., Feng L., Pelicano H., Wang H., Keating M.J. (2012). Novel role of NOX in supporting aerobic glycolysis in cancer cells with mitochondrial dysfunction and as a potential target for cancer therapy. PLoS Biol.

[79] Lin C.C., Cheng T.L., Tsai W.H., Tsai H.J., Hu K.H., Chang H.C., Yeh C.W., Chen Y.C., Liao C.C., Chang W.T. (2012). Loss of the respiratory enzyme citrate synthase directly links the Warburg effect to tumor malignancy. Sci. Rep.

[80] Singh M., Singh V.N., August J.T., Horecker B.L. (1974). Alterations in glucose metabolism in chick embryo cells transformed by Rous sarcoma virus. Transformation-specific changes in the activities of key enzymes of the glycolytic and hexose monophosphate shunt pathways. Arch. Biochem. Biophys..

[81] Darekar S., Georgiou K., Yurchenko M., Yenamandra S.P., Chachami G., Simos G., Klein G., Kashuba E. (2012). Epstein-Barr virus immortalization of human B-cells leads to stabilization of hypoxia-induced factor 1 alpha, congruent with the Warburg effect. PLoS One.

[82] Cole M.A., Crawford D.W., Warner N.E., Puffer H.W. (1983). Correlation of regional disease and *in vivo* PO2 in rat mammary adenocarcinoma. Am. J. Pathol.

[83] Joyce R.M., Vincent P.C. (1983). Advantage of reduced oxygen tension in growth of human melanomas in semi-solid cultures: Quantitative analysis. Br. J. Cancer.

[84] Ezashi T., Das P., Roberts R.M. (2005). Low O_2_ tensions and the prevention of differentiation of hES cells. Proc. Natl. Acad. Sci. USA.

[85] Yoshida Y., Takahashi K., Okita K., Ichisaka T., Yamanaka S. (2009). Hypoxia enhances the generation of induced pluripotent stem cells. Cell Stem Cell.

[86] Brizel D.M., Sibley G.S., Prosnitz L.R., Scher R.L., Dewhirst M.W. (1997). Tumor hypoxia adversely affects the prognosis of carcinoma of the head and neck. Int. J. Radiat. Oncol. Biol. Phys.

[87] Nozue M., Lee I., Yuan F., Teicher B.A., Brizel D.M., Dewhirst M.W., Milross C.G., Milas L., Song C.W., Thomas C.D. (1997). Interlaboratory variation in oxygen tension measurement by Eppendorf “Histograph” and comparison with hypoxic marker. J. Surg. Oncol.

[88] Nordsmark M., Høyer M., Keller J., Nielsen O.S., Jensen O.M., Overgaard J. (1996). The relationship between tumor oxygenation and cell proliferation in human soft tissue sarcomas. Int. J. Radiat. Oncol. Biol. Phys.

[89] Sundfør K., Lyng H., Rofstad E.K. (1998). Tumour hypoxia and vascular density as predictors of metastasis in squamous cell carcinoma of the uterine cervix. Br. J. Cancer.

[90] Nordsmark M., Overgaard J. (2000). A confirmatory prognostic study on oxygenation status and loco-regional control in advanced head and neck squamous cell carcinoma treated by radiation therapy. Radiother. Oncol.

[91] Young S.D., Marshall R.S., Hill R.P. (1988). Hypoxia induces DNA overreplication and enhances metastatic potential of murine tumor cells. Proc. Natl. Acad. Sci. USA.

[92] Young S.D., Hill R.P. (1990). Effects of reoxygenation on cells from hypoxic regions of solid tumors: Anticancer drug sensitivity and metastatic potential. J. Natl. Cancer Inst.

[93] Rofstad E.K., Måseide K. (1999). Radiobiological and immunohistochemical assessment of hypoxia in human melanoma xenografts: Acute and chronic hypoxia in individual tumours. Int. J. Radiat. Biol.

[94] Olsen D.R., Singstad T.E., Rofstad E.K. (1999). Effects of hyperthermia on bioenergetic status and phosphorus T1S in human melanoma xenografts monitored by 31P-MRS. Magn. Reson. Imaging.

[95] Stackpole C.W., Groszek L., Kalbag S.S. (1994). Benign-to-malignant B16 melanoma progression induced in two stages *in vitro* by exposure to hypoxia. J. Natl. Cancer Inst.

[96] Graham C.H., Forsdike J., Fitzgerald C.J., Macdonald-Goodfellow S. (1999). Hypoxia-mediated stimulation of carcinoma cell invasiveness via upregulation of urokinase receptor expression. Int. J. Cancer.

[97] Ebos J.M., Lee C.R., Cruz-Munoz W., Bjarnason G.A., Christensen J.G., Kerbel R.S. (2009). Accelerated metastasis after short-term treatment with a potent inhibitor of tumor angiogenesis. Cancer Cell.

[98] Pfeiffer T., Schuster S., Bonhoeffer S. (2001). Cooperation and competition in the evolution of ATP-producing pathways. Science.

[99] DeBerardinis R.J., Mancuso A., Daikhin E., Nissim I., Yudkoff M., Wehrli S., Thompson C.B. (2007). Beyond aerobic glycolysis: Transformed cells can engage in glutamine metabolism that exceeds the requirement for protein and nucleotide synthesis. Proc. Natl. Acad. Sci. USA.

[100] Cairns R.A., Kalliomaki T., Hill R.P. (2001). Acute (cyclic) hypoxia enhances spontaneous metastasis of KHT murine tumors. Cancer Res.

[101] Knudson A.G. (2001). Two genetic hits (more or less) to cancer. Nat. Rev. Cancer.

[102] Bertram J.S. (2000). The molecular biology of cancer. Mol. Aspects Med.

[103] Roach J.C., Glusman G., Smit A.F., Huff C.D., Hubley R., Shannon P.T., Rowen L., Pant K.P., Goodman N., Bamshad M. (2010). Analysis of genetic inheritance in a family quartet by whole-genome sequencing. Science.

[104] Hitchler M.J., Domann F.E. (2009). Metabolic defects provide a spark for the epigenetic switch in cancer. Free Radic. Biol. Med.

[105] Hitchler M.J., Domann F.E. (2012). Redox regulation of the epigenetic landscape in cancer: A role for metabolic reprogramming in remodeling the epigenome. Free Radic. Biol. Med.

[106] Gambini J., Gomez-Cabrera M.C., Borras C., Valles S.L., Lopez-Grueso R., Martinez-Bello V.E., Herranz D., Pallardo F.V., Tresguerres J.A., Serrano M. (2011). Free [NADH]/[NAD(+)] regulates sirtuin expression. Arch. Biochem. Biophys.

[107] Van Horssen R., Willemse M., Haeger A., Attanasio F., Güneri T., Schwab A., Stock C.M., Buccione R., Fransen J.A., Wieringa B. (2013). Intracellular NAD(H) levels control motility and invasion of glioma cells. Cell Mol. Life Sci..

[108] Mato J.M., Lu S.C. (2011). The hepatocarcinogenic effect of methionine and choline deficient diets: An adaptation to the Warburg effect?. Alcohol Clin. Exp. Res.

[109] Mato J.M., Lu S.C. (2012). S-adenosylmethionine in liver health, injury, and cancer. Physiol. Rev.

[110] Watson W.H., Song Z., Kirpich I.A., Deaciuc I.V., Chen T., McClain C.J. (2011). Ethanol exposure modulates hepatic S-adenosylmethionine and S-adenosylhomocysteine levels in the isolated perfused rat liver through changes in the redox state of the NADH/NAD(+) system. Biochim. Biophys. Acta.

[111] Yang W., Zheng Y., Xia Y., Ji H., Chen X., Guo F., Lyssiotis C.A., Aldape K., Cantley L.C., Lu Z. (2012). ERK1/2-dependent phosphorylation and nuclear translocation of PKM2 promotes the Warburg effect. Nat. Cell Biol.

[112] Neary C.L., Pastorino J.G. (2010). Nucleocytoplasmic shuttling of hexokinase II in a cancer cell. Biochem. Biophys. Res. Commun.

[113] Peláez R., Herrero P., Moreno F. (2009). Nuclear export of the yeast hexokinase 2 protein requires the Xpo1 Crm1-dependent pathway. J. Biol. Chem.

[114] Friis R.M., Wu B.P., Reinke S.N., Hockman D.J., Sykes B.D., Schultz M.C. (2009). A glycolytic burst drives glucose induction of global histone acetylation by picNuA4 and SAGA. Nucleic. Acids Res.

[115] Daran-Lapujade P., Rossell S., van Gulik W.M., Luttik M.A., de Groot M.J., Slijper M., Heck A.J., Daran J.M., de Winde J.H., Westerhoff H.V. (2007). The fluxes through glycolytic enzymes in Saccharomyces cerevisiae are predominantly regulated at posttranscriptional levels. Proc. Natl. Acad. Sci. USA.

[116] Chen Z., Odstrcil E.A., Tu B.P., McKnight S.L. (2007). Restriction of DNA replication to the reductive phase of the metabolic cycle protects genome integrity. Science.

[117] Hilton J., Walker M.D. (1977). DNA strand scission and its repair following exposure of cells to inhibitors of oxidative phosphorylation. Biochem. Biophys. Res. Commun.

[118] Fry D.W. (1990). Cytotoxic synergism between trimetrexate and etoposide. Evidence that trimetrexate potentiates etoposide-induced protein-associated DNA strand breaks in L1210 leukemia cells through alterations in intracellular ATP concentrations. Biochem. Pharmacol.

[119] Dalrymple G.V., Sanders J.L., Baker M.L., Wilkinson K.P. (1969). The effect of 2,4-dinitrophenol on the repair of radiation injury by L-cells. Radiat. Res.

[120] Moss A.J., Dalrymple G.V., Sanders J.L., Wilkinson K.P., Nash J.C. (1971). Dinitrophenol inhibits the rejoining of radiation-induced DNA breaks by L-cells. Biophys. J.

[121] Wang Z., Wu X., Friedberg E.C. (1993). Nucleotide-excision repair of DNA in cell-free extracts of the yeast Saccharomyces cerevisiae. Proc. Natl. Acad. Sci. USA.

[122] Lagostera M., Guerrero R., Villaverde A., Barbé J. (1985). Effect of adenine, cytidine and guanosine on the expression of the SOS system in *Escherichia coli*. J. Gen. Microbiol.

[123] Thomas D.C., Roberts J.D., Kunkel T.A. (1991). Heteroduplex repair in extracts of human HeLa cells. J. Biol. Chem.

[124] Rapaport E., Garcia-Blanco M.A., Zamecnik P.C. (1979). Regulation of DNA replication in S phase nuclei by ATP and ADP pools. Proc. Natl. Acad. Sci. USA.

[125] Laureti L., Selva M., Dairou J., Matic I. (2013). Reduction of dNTP levels enhances DNA replication fidelity *in vivo*. DNA Repair.

[126] Wijker J.E., Jensen P.R., Snoep J.L., vaz Gomes A., Guiral M., Jongsma A.P., de Waal A., Hoving S., van Dooren S., van der Weijden C.C. (1995). Energy, control and DNA structure in the living cell. Biophys. Chem.

[127] Singleton M.R., Wigley D.B. (2003). Multiple roles for ATP hydrolysis in nucleic acid modifying enzymes. EMBO J.

[128] Okorokov A.L., Milner J. (1999). An ATP/ADP-dependent molecular switch regulates the stability of p53-DNA complexes. Mol. Cell Biol.

[129] Yang J.G., Narlikar G.J. (2007). FRET-based methods to study ATP-dependent changes in chromatin structure. Methods.

[130] Varga-Weisz P. (2001). ATP-dependent chromatin remodeling factors: Nucleosome shufflers with many missions. Oncogene.

[131] Laval F. (1980). Effect of uncouplers on radiosensitivity and mutagenicity in x-irradiated mammalian cells. Proc. Natl. Acad. Sci. USA.

[132] Tahanian E., Peiro S., Annabi B. (2011). Low intracellular ATP levels exacerbate carcinogen-induced inflammatory stress response and inhibit *in vitro* tubulogenesis in human brain endothelial cells. J. Inflamm. Res.

[133] Jonson I., Ougland R., Klungland A., Larsen E. (2013). Oxidative stress causes DNA triplet expansion in Huntington’s disease mouse embryonic stem cells. Stem Cell Res.

[134] Zhang M., Yang C., Liu H., Sun Y. (2013). Induced pluripotent stem cells are sensitive to DNA damage. Genomics Proteomics Bioinform.

[135] Tanori M., Pasquali E., Leonardi S., Casciati A., Giardullo P., de Stefano I., Mancuso M., Saran A., Pazzaglia S. (2013). Developmental and oncogenic radiation effects on neural stem cells and their differentiating progeny in mouse cerebellum. Stem Cells.

[136] Nouspikel T. (2013). Genetic instability in human embryonic stem cells: Prospects and caveats. Future Oncol.

[137] Kenyon J., Gerson S.L. (2007). The role of DNA damage repair in aging of adult stem cells. Nucleic Acids Res.

[138] Morley A., Seshadri R., Trainor K., Sorrell J. (1978). Is aplastic anaemia due to abnormality of DNA?. Lancet.

[139] Gerson S.L., Trey J.E., Miller K., Benjamin E. (1987). Repair of O6-alkylguanine during DNA synthesis in murine bone marrow hematopoietic precursors. Cancer Res.

[140] Luo L.Z., Gopalakrishna-Pillai S., Nay S.L., Park S.W., Bates S.E., Zeng X., Iverson L.E., O’Connor T.R. (2012). DNA repair in human pluripotent stem cells is distinct from that in non-pluripotent human cells. PLoS One.

[141] Cousin W., Ho M.L., Desai R., Tham A., Chen R.Y., Kung S., Elabd C., Conboy I.M. (2013). Regenerative capacity of old muscle stem cells declines without significant accumulation of DNA damage. PLoS One.

[142] Pavlides S., Whitaker-Menezes D., Castello-Cros R., Flomenberg N., Witkiewicz A.K., Frank P.G., Casimiro M.C., Wang C., Fortina P., Addya S. (2009). The reverse Warburg effect: Aerobic glycolysis in cancer associated fibroblasts and the tumor stroma. Cell Cycle.

[143] Sloan E.K., Ciocca D.R., Pouliot N., Natoli A., Restall C., Henderson M.A., Fanelli M.A., Cuello-Carrión F.D., Gago F.E., Anderson R.L. (2009). Stromal cell expression of caveolin-1 predicts outcome in breast Cancer. Am. J. Pathol.

[144] Whitaker-Menezes D., Martinez-Outschoorn U.E., Lin Z., Ertel A., Flomenberg N., Witkiewicz A.K., Birbe R.C., Howell A., Pavlides S., Gandara R. (2011). Evidence for a stromal-epithelial “lactate shuttle” in human tumors: MCT4 is a marker of oxidative stress in cancer-associated fibroblasts. Cell Cycle.

[145] Balliet R.M., Capparelli C., Guido C., Pestell T.G., Martinez-Outschoorn U.E., Lin Z., Whitaker-Menezes D., Chiavarina B., Pestell R.G., Howell A. (2011). Mitochondrial oxidative stress in cancer-associated fibroblasts drives lactate production: Promoting breast cancer tumor growth, understanding the aging and cancer connection. Cell Cycle.

[146] Samudio I., Fiegl M., McQueen T., Clise-Dwyer K., Andreeff M. (2008). The warburg effect in leukemia-stroma cocultures is mediated by mitochondrial uncoupling associated with uncoupling protein 2 activation. Cancer Res.

[147] Konopleva M., Andreeff M. (2007). Targeting the leukemia microenvironment. Curr. Drug Targets.

[148] Wang L., O’Leary H., Fortney J., Gibson L.F. (2007). Ph+/VE-cadherin+ identifies a stem cell like population of acute lymphoblastic leukemia sustained by bone marrow niche cells. Blood.

[149] Echtay K.S., Murphy M.P., Smith R.A., Talbot D.A., Brand M.D. (2002). Superoxide activates mitochondrial uncoupling protein 2 from the matrix side. Studies using targeted antioxidants. J. Biol. Chem.

[150] Samudio I., Fiegl M., Andreeff M. (2009). Mitochondrial uncoupling and the Warburg effect: Molecular basis for the reprogramming of cancer cell metabolism. Cancer Res.

[151] Pecqueur C., Bui T., Gelly C., Hauchard J., Barbot C., Bouillaud F., Ricquier D., Miroux B., Thompson C.B. (2008). Uncoupling protein-2 controls proliferation by promoting fatty acid oxidation and limiting glycolysis-derived pyruvate utilization. FASEB J.

[152] Harper M.E., Antoniou A., Villalobos-Menuey E., Russo A., Trauger R., Vendemelio M., George A., Bartholomew R., Carlo D., Shaikh A. (2002). Characterization of a novel metabolic strategy used by drug-resistant tumor cells. FASEB J.

[153] Mattiasson G., Shamloo M., Gido G., Mathi K., Tomasevic G., Yi S., Warden C.H., Castilho R.F., Melcher T., Gonzalez-Zulueta M. (2003). Uncoupling protein-2 prevents neuronal death and diminishes brain dysfunction after stroke and brain trauma. Nat. Med.

[154] Mintz B., Illmensee K. (1975). Normal genetically mosaic mice produced from malignant teratocarcinoma cells. Proc. Natl. Acad. Sci. USA.

[155] Ruckenstuhl C., Büttner S., Carmona-Gutierrez D., Eisenberg T., Kroemer G., Sigrist S.J., Fröhlich K.U., Madeo F. (2009). The Warburg effect suppresses oxidative stress induced apoptosis in a yeast model for Cancer. PLoS One.

[156] Eguchi Y., Shimizu S., Tsujimoto Y. (1997). Intracellular ATP levels determine cell death fate by apoptosis or necrosis. Cancer Res.

[157] Eguchi Y., Srinivasan A., Tomaselli K.J., Shimizu S., Tsujimoto Y. (1999). ATP-dependent steps in apoptotic signal transduction. Cancer Res.

[158] Shen J., Liu X., Yu W.M., Liu J., Nibbelink M.G., Guo C., Finkel T., Qu C.K. (2011). A critical role of mitochondrial phosphatase Ptpmt1 in embryogenesis reveals a mitochondrial metabolic stress-induced differentiation checkpoint in embryonic stem cells. Mol. Cell Biol.

[159] Lane N., Martin W. (2010). The energetics of genome complexity. Nature.

[160] Navarro A., Boveris A. (2009). Brain mitochondrial dysfunction and oxidative damage in Parkinson’s disease. J. Bioenerg. Biomembr.

[161] Balaban R.S., Nemoto S., Finkel T. (2005). Mitochondria, oxidants, and aging. Cell.

[162] Lonergan T., Brenner C., Bavister B. (2006). Differentiation-related changes in mitochondrial properties as indicators of stem cell competence. J. Cell Physiol.

[163] Brown G.C. (1992). Control of respiration and ATP synthesis in mammalian mitochondria and cells. Biochem. J.

[164] Thundathil J., Filion F., Smith L.C. (2005). Molecular control of mitochondrial function in preimplantation mouse embryos. Mol. Reprod. Dev.

[165] Van Blerkom J. (2009). Mitochondria in early mammalian development. Semin. Cell Dev. Biol.

[166] St John J.C., Ramalho-Santos J., Gray H.L., Petrosko P., Rawe V.Y., Navara C.S., Simerly C.R., Schatten G.P. (2005). The expression of mitochondrial DNA transcription factors during early cardiomyocyte *in vitro* differentiation from human embryonic stem cells. Cloning Stem Cells.

[167] St John J. (2014). The control of mtDNA replication during differentiation and development. Biochim. Biophys. Acta.

[168] Holley A.K., Dhar S.K., Xu Y., St Clair D.K. (2012). Manganese superoxide dismutase: Beyond life and death. Amino Acids.

[169] Duttaroy A., Paul A., Kundu M., Belton A. (2003). A SOD2 null mutation confers severely reduced adult life span in Drosophila. Genetics.

[170] Van Remmen H., Ikeno Y., Hamilton M., Pahlavani M., Wolf N., Thorpe S.R., Alderson N.L., Baynes J.W., Epstein C.J., Huang T.T. (2003). Life-long reduction in MnSOD activity results in increased DNA damage and higher incidence of cancer but does not accelerate aging. Physiol. Genome.

[171] Olivotto M., Arcangeli A., Caldini R., Chevanne M., Cipolleschi M.G., Dello Sbarba P. (1984). Metabolic aspects of cell cycle regulation in normal and cancer cells. Toxicol. Pathol.

[172] Li W., Nichols K., Nathan C.A., Zhao Y. (2013). Mitochondrial uncoupling protein 2 is up-regulated in human head and neck, skin, pancreatic, and prostate tumors. Cancer Biomark.

[173] Kuai X.Y., Ji Z.Y., Zhang H.J. (2010). Mitochondrial uncoupling protein 2 expression in colon cancer and its clinical significance. World J. Gastroenterol.

[174] Zhang G., Qu Y., Dang S., Yang Q., Shi B., Hou P. (2013). Variable copy number of mitochondrial DNA (mtDNA) predicts worse prognosis in advanced gastric cancer patients. Diagn. Pathol.

[175] Criscuolo F., Mozo J., Hurtaud C., Nübel T., Bouillaud F. (2006). UCP2, UCP3, avUCP, what do they do when proton transport is not stimulated? Possible relevance to pyruvate and glutamine metabolism. Biochim. Biophys. Acta.

[176] Zhang J., Khvorostov I., Hong J.S., Oktay Y., Vergnes L., Nuebel E., Wahjudi P.N., Setoguchi K., Wang G., Do A. (2011). UCP2 regulates energy metabolism and differentiation potential of human pluripotent stem cells. EMBO J.

[177] Simonnet H., Alazard N., Pfeiffer K., Gallou C., Béroud C., Demont J., Bouvier R., Schägger H., Godinot C. (2002). Low mitochondrial respiratory chain content correlates with tumor aggressiveness in renal cell carcinoma. Carcinogenesis.

[178] Wallace D.C. (2012). Mitochondria and Cancer. Nat. Rev. Cancer.

[179] Pokorný J., Jandová A., Nedbalová M., Jelínek F., Cifra M., Kučera O., Havelka D., Vrba J., Vrba J., Coček A. (2012). Mitochondrial metabolism—Neglected link of cancer transformation and treatment. Prague Med. Rep.

[180] Dang C.V. (2012). Links between metabolism and Cancer. Genes Dev.

[181] Butow R.A., Avadhani N.G. (2004). Mitochondrial signaling: The retrograde response. Mol. Cell.

[182] Miceli M.V., Jazwinski S.M. (2005). Nuclear gene expression changes due to mitochondrial dysfunction in ARPE-19 cells: Implications for age-related macular degeneration. Investig. Ophthalmol. Vis. Sci.

[183] Jazwinski S.M. (2005). The retrograde response links metabolism with stress responses, chromatin-dependent gene activation, and genome stability in yeast aging. Gene.

[184] Singh K.K., Kulawiec M., Still I., Desouki M.M., Geradts J., Matsui S. (2005). Inter-genomic cross talk between mitochondria and the nucleus plays an important role in tumorigenesis. Gene.

[185] Traven A., Wong J.M., Xu D., Sopta M., Ingles C.J. (2001). Interorganellar communication. Altered nuclear gene expression profiles in a yeast mitochondrial dna mutant. J. Biol. Chem.

[186] Veatch J.R., McMurray M.A., Nelson Z.W., Gottschling D.E. (2009). Mitochondrial dysfunction leads to nuclear genome instability via an iron-sulfur cluster defect. Cell.

[187] Erol A. (2005). Retrograde regulation due to mitochondrial dysfunction may be an important mechanism for carcinogenesis. Med. Hypotheses.

[188] Kyle J.L., Riesen W.H. (1970). Stress and cigarette smoke effects on lung mitochondrial phosphorylation. Arch. Environ. Health.

[189] Hoffmann R.F., Zarrintan S., Brandenburg S.M., Kol A., de Bruin H.G., Jafari S., Dijk F., Kalicharan D., Kelders M., Gosker H.R. (2013). Prolonged cigarette smoke exposure alters mitochondrial structure and function in airway epithelial cells. Respir. Res.

[190] Sanchez-Alvarez R., Martinez-Outschoorn U.E., Lin Z., Lamb R., Hulit J., Howell A., Sotgia F., Rubin E., Lisanti M.P. (2013). Ethanol exposure induces the cancer-associated fibroblast phenotype and lethal tumor metabolism: Implications for breast cancer prevention. Cell Cycle.

[191] Kiessling K.H., Pilström L. (1966). Effect of ethanol on rat liver. I. Enzymatic and histological studies of liver mitochondria. Q. J. Stud. Alcohol.

[192] Syed M., Skonberg C., Hansen S.H. (2013). Effect of some organic solvents on oxidative phosphorylation in rat liver mitochondria: Choice of organic solvents. Toxicol. in Vitro.

[193] Hadler H.I., Mueller K.W. (1978). The disturbance of oxidative phosphorylation in rat liver mitochondria by the carcinogens 12-hydroxystearic acid and its methyl ester. J. Environ. Pathol. Toxicol.

[194] Parkin D.M. (2006). The global health burden of infection-associated cancers in the year 2002. Int. J. Cancer.

[195] Koike K. (2009). Hepatitis B virus X gene is implicated in liver carcinogenesis. Cancer Lett.

[196] Clippinger A.J., Bouchard M.J. (2008). Hepatitis B virus HBx protein localizes to mitochondria in primary rat hepatocytes and modulates mitochondrial membrane potential. J. Virol.

[197] D’Agostino D.M., Bernardi P., Chieco-Bianchi L., Ciminale V. (2005). Mitochondria as functional targets of proteins coded by human tumor viruses. Adv. Cancer Res.

[198] Koura M., Isaka H., Yoshida M.C., Tosu M., Sekiguchi T. (1982). Suppression of tumorigenicity in interspecific reconstituted cells and cybrids. Gann.

[199] Israel B.A., Schaeffer W.I. (1987). Cytoplasmic suppression of malignancy. In Vitro Cell Dev. Biol.

[200] Howell A.N., Sager R. (1978). Tumorigenicity and its suppression in cybrids of mouse and Chinese hamster cell lines. Proc. Natl. Acad. Sci. USA.

[201] McKinnell R.G., Deggins B.A., Labat D.D. (1969). Transplantation of pluripotential nuclei from triploid frog tumors. Science.

[202] Li L., Connelly M.C., Wetmore C., Curran T., Morgan J.I. (2003). Mouse embryos cloned from brain tumors. Cancer Res.

[203] Hochedlinger K., Blelloch R., Brennan C., Yamada Y., Kim M., Chin L., Jaenisch R. (2004). Reprogramming of a melanoma genome by nuclear transplantation. Genes Dev.

[204] Petros J.A., Baumann A.K., Ruiz-Pesini E., Amin M.B., Sun C.Q., Hall J., Lim S., Issa M.M., Flanders W.D., Hosseini S.H. (2005). mtDNA mutations increase tumorigenicity in prostate cancer. Proc. Natl. Acad. Sci. USA.

[205] Wang T., Marquardt C., Foker J. (1976). Aerobic glycolysis during lymphocyte proliferation. Nature.

[206] Brand K., Aichinger S., Forster S., Kupper S., Neumann B., Nürnberg W., Ohrisch G. (1988). Cell–cycle-related metabolic and enzymatic events in proliferating rat thymocytes. Eur. J. Biochem.

[207] Siverio J.M., Torres N.V., Meléndez-Hevia E. (1985). Activities of l-lactate and glycerol phosphate production rates *in vitro* from glucose 6-phosphate in regenerating rat liver. Int. J. Biochem.

[208] Forni E., Filipazzi A. (1964). Study of some aspects of liver regeneration after partial hepatectomy. I. Oxigen consumption and glycolysis of regenerating tissue. Chir. Patol. Sper.

[209] Menyhárt J., Horváth A., Rosta A. (1971). A reliable index of tissue metabolic activity during the initial phase of rat liver regeneration. Acta Biochim. Biophys. Acad. Sci. Hung.

[210] Vihersaari T., Kivisaari J., Ninikoski J. (1979). Effect of changes in inspired oxygen tension on wound metabolism. Ann. Surg.

[211] Braskén P., Renvall S. (1990). Local energy metabolism in healing colon anastomosis. An enzyme-histochemical study in rats. Acta Chir. Scand.

[212] O’Connor R.J. (1950). The effect on cell division of inhibiting aerobic glycolysis. Br. J. Exp. Pathol.

[213] Bax B.E., Bloxam D.L. (1997). Energy metabolism and glycolysis in human placental trophoblast cells during differentiation. Biochim. Biophys. Acta.

[214] Kwon H.J. (2013). ATP oscillations mediate inductive action of FGF and SHH signalling on prechondrogenic condensation. Cell Biochem. Funct.

[215] Tsuchiya M., Ross J. (2003). Advantages of external periodic events to the evolution of biochemical oscillatory reactions. Proc. Natl. Acad. Sci. USA.

[216] Boiteux A., Goldbeter A., Hess B. (1975). Control of oscillating glycolysis of yeast by stochastic, periodic, and steady source of substrate: A model and experimental study. Proc. Natl. Acad. Sci. USA.

[217] Yang J.H., Yang L., Qu Z., Weiss J.N. (2008). Glycolytic oscillations in isolated rabbit ventricular myocytes. J. Biol. Chem.

[218] Agathocleous M., Love N.K., Randlett O., Harris J.J., Liu J., Murray A.J., Harris W.A. (2012). Metabolic differentiation in the embryonic retina. Nat. Cell Biol.

[219] Ho J., de Moura M.B., Lin Y., Vincent G., Thorne S., Duncan L.M., Hui-Min L., Kirkwood J.M., Becker D., van Houten B. (2012). Importance of glycolysis and oxidative phosphorylation in advanced melanoma. Mol. Cancer.

[220] Maglietta R., Liuzzi V.C., Cattaneo E., Laczko E., Piepoli A., Panza A., Carella M., Palumbo O., Staiano T., Buffoli F. (2012). Molecular pathways undergoing dramatic transcriptomic changes during tumor development in the human colon. BMC Cancer.

[221] Brown N.J., Higham S.E., Perunovic B., Arafa M., Balasubramanian S., Rehman I. (2013). Lactate dehydrogenase-B is silenced by promoter methylation in a high frequency of human breast cancers. PLoS One.

[222] Kim S., Kim do H., Jung W.H., Koo J.S. (2012). Metabolic phenotypes in triple-negative breast Cancer. Tumour Biol.

[223] Yadava N., Schneider S.S., Jerry D.J., Kim C. (2013). Impaired mitochondrial metabolism and mammary carcinogenesis. J. Mammary Gland Biol. Neoplasia.

[224] Buravkova L.B., Rylova Y.V., Andreeva E.R., Kulikov A.V., Pogodina M.V., Zhivotovsky B., Gogvadze V. (2013). Low ATP level is sufficient to maintain the uncommitted state of multipotent mesenchymal stem cells. Biochim. Biophys. Acta.

[225] Xun Z., Lee D.Y., Lim J., Canaria C.A., Barnebey A., Yanonne S.M., McMurray C.T. (2012). Retinoic acid-induced differentiation increases the rate of oxygen consumption and enhances the spare respiratory capacity of mitochondria in SH-SY5Y cells. Mech. Ageing Dev.

[226] Saumet A., Vetter G., Bouttier M., Antoine E., Roubert C., Orsetti B., Theillet C., Lecellier C.H. (2012). Estrogen and retinoic acid antagonistically regulate several microRNA genes to control aerobic glycolysis in breast cancer cells. Mol. Biosyst.

[227] Heinrich R., Meléndez-Hevia E., Montero F., Nuño J.C., Stephani A., Waddell T.G. (1999). The structural design of glycolysis: An evolutionary approach. Biochem. Soc. Trans.

[228] Van Dijken J.P., Weusthuis R.A., Pronk J.T. (1993). Kinetics of growth and sugar consumption in yeasts. Antonie Van Leeuwenhoek.

[229] Inderlied C.B., Sypherd P.S. (1978). Glucose metabolism and dimorphism in Mucor. J. Bacteriol.

[230] Poolman B. (1993). Energy transduction in lactic acid bacteria. FEMS Microbiol. Rev.

[231] Veech R.L., Kashiwaya Y., Gates D.N., King M.T., Clarke K. (2002). The energetics of ion distribution: The origin of the resting electric potential of cells. IUBMB Life.

[232] Veech R.L., Kashiwaya Y., King M.T. (1995). The resting membrane potential of cells are measures of electrical work, not of ionic currents. Integr. Physiol. Behav. Sci.

[233] Masuda T., Dobson G.P., Veech R.L. (1990). The Gibbs-Donnan near-equilibrium system of heart. J. Biol. Chem.

[234] Racker E. (1976). Why do tumor cells have a high aerobic glycolysis?. J. Cell Physiol.

[235] Schmidt H., Siems W., Müller M., Dumdey R., Rapoport S.M. (1991). ATP-producing and consuming processes of Ehrlich mouse ascites tumor cells in proliferating and resting phases. Exp. Cell Res.

[236] Termonia Y., Ross J. (1981). Oscillations and control features in glycolysis: Numerical analysis of a comprehensive model. Proc. Natl. Acad. Sci. USA.

[237] Ganitkevich V., Mattea V., Benndorf K. (2010). Glycolytic oscillations in single ischemic cardiomyocytes at near anoxia. J. Gen. Physiol.

[238] Ahsan H., Halpern J., Kibriya M.G., Pierce B.L., Tong L., Gamazon E., McGuire V., Felberg A., Shi J., Jasmine F. (2014). A Genome-wide association study of early-onset breast cancer identifies PFKM as a novel breast cancer gene and supports a common genetic spectrum for breast cancer at any age. Cancer Epidemiol. Biomark. Prev.

[239] Klarer A.C., O’Neal J., Imbert-Fernandez Y., Clem A., Ellis S.R., Clark J., Clem B., Chesney J., Telang S. (2014). Inhibition of 6-phosphofructo-2-kinase (PFKFB3) induces autophagy as a survival mechanism. Cancer Metab.

[240] Goldbeter A. (2013). Oscillatory enzyme reactions and Michaelis-Menten kinetics. FEBS Lett.

[241] Mahnensmith R.L., Aronson P.S. (1985). The plasma membrane sodium-hydrogen exchanger and its role in physiological and pathophysiological processes. Circ. Res.

[242] Sennoune S.R., Bakunts K., Martínez G.M., Chua-Tuan J.L., Kebir Y., Attaya M.N., Martínez-Zaguilán R. (2004). Vacuolar H^+^-ATPase in human breast cancer cells with distinct metastatic potential: Distribution and functional activity. Am. J. Physiol. Cell Physiol.

[243] Baluch S., Midwood C.J., Griffiths J.R., Stubbs M., Coombes R.C. (1991). Monitoring therapeutic response to tamoxifen in NMU-induced rat mammary tumours by 31P MRS. Br. J. Cancer.

[244] Navon G., Ogawa S., Shulman R.G., Yamane T. (1977). 31P nuclear magnetic resonance studies of Ehrlich ascites tumor cells. Proc. Natl. Acad. Sci. USA.

[245] Ross B.D., Higgins R.J., Boggan J.E., Knittel B., Garwood M. (1988). 31P NMR spectroscopy of the *in vivo* metabolism of an intracerebral glioma in the rat. Magn. Reson. Med.

[246] Bradbury D.A., Simmons T.D., Slater K.J., Crouch S.P. (2000). Measurement of the ADP:ATP ratio in human leukaemic cell lines can be used as an indicator of cell viability, necrosis and apoptosis. J. Immunol. Methods.

[247] Singer S., Souza K., Thilly W.G. (1995). Pyruvate utilization, phosphocholine and adenosine triphosphate (ATP) are markers of human breast tumor progression: A 31P- and 13C- nuclear magnetic resonance (NMR) spectroscopy study. Cancer Res.

[248] Rizwan A., Serganova I., Khanin R., Karabeber H., Ni X., Thakur S., Zakian K.L., Blasberg R., Koutcher J.A. (2013). Relationships between LDH-A, lactate, and metastases in 4T1 breast tumors. Clin. Cancer Res.

[249] Park J.M., Park J.H. (2001). Human *in vivo* 31P MR spectroscopy of benign and malignant breast tumors. Korean J. Radiol.

[250] Golinska M., Troy H., Chung Y.L., McSheehy P.M., Mayr M., Yin X., Ly L., Williams K.J., Airley R.E., Harris A.L. (2011). Adaptation to HIF-1 deficiency by upregulation of the AMP/ATP ratio and phosphofructokinase activation in hepatomas. BMC Cancer.

[251] Cho Y.M., Kwon S., Pak Y.K., Seol H.W., Choi Y.M., Park D.J., Park K.S., Lee H.K. (2006). Dynamic changes in mitochondrial biogenesis and antioxidant enzymes during the spontaneous differentiation of human embryonic stem cells. Biochem. Biophys. Res. Commun.

[252] Lonergan T., Bavister B., Brenner C. (2007). Mitochondria in stem cells. Mitochondrion.

[253] Hoffer F.A., Taylor G.A., Spevak M., Ingber D., Fenton T. (1989). Metabolism of tumor regression from angiogenesis inhibition: 31P magnetic resonance spectroscopy. Magn. Reson. Med.

[254] Vlashi E., Lagadec C., Vergnes L., Matsutani T., Masui K., Poulou M., Popescu R., della Donna L., Evers P., Dekmezian C. (2011). Metabolic state of glioma stem cells and nontumorigenic cells. Proc. Natl. Acad. Sci. USA.

[255] Frieden B.R., Gatenby R.A. (2011). Information dynamics in living systems, prokaryotes: Eukaryotes, and cancer. PLoS One.

[256] Teschendorff A.E., Severini S. (2010). Increased entropy of signal transduction in the cancer metastasis phenotype. BMC Syst. Biol.

[257] West J., Lacasa L., Severini S., Teschendorff A. (2012). Approximate entropy of network parameters. Phys. Rev. E Stat. Nonlin. Soft Matter Phys.

[258] Banerji C.R., Severini S., Teschendorff A.E. (2013). Network transfer entropy and metric space for causality inference. Phys. Rev. E Stat. Nonlin. Soft Matter Phys.

[259] Banerji C.R., Miranda-Saavedra D., Severini S., Widschwendter M., Enver T., Zhou J.X., Teschendorff A.E. (2013). Cellular network entropy as the energy potential in Waddington's differentiation landscape. Sci. Rep.

[260] Ozernyuk N.D., Zotin A.I., Yurowitzky Y.G. (1973). Deviation of the living system from the stationary state during oogenesis. Wilhelm Roux Archiv.

[261] Zotin A.A., Zotin A.I. (1997). Phenomenological theory of ontogenesis. Int. J. Dev. Biol.

[262] Martyushev L.M. (2013). Entropy and entropy production: Old misconceptions and new breakthroughs. Entropy.

[263] Kumar P., Rajput S., Verma A., De S., Datta T.K. (2013). Expression pattern of glucose metabolism genes in relation to development rate of buffalo (*Bubalus bubalis*) oocytes and *in vitro*-produced embryos. Theriogenology.

[264] Sugimura S., Matoba S., Hashiyada Y., Aikawa Y., Ohtake M., Matsuda H., Kobayashi S., Konishi K., Imai K. (2012). Oxidative phosphorylation-linked respiration in individual bovine oocytes. J. Reprod. Dev.

[265] Abele D. (2002). Toxic oxygen: The radical life-giver. Nature.

[266] Houghton J., Morozov A., Smirnova I., Wang T.C. (2007). Stem cells and Cancer. Semin. Cancer Biol.

[267] DePinho R.A. (2000). The age of cancer. Nature.

[268] Armitage P., Doll R. (2004). The age distribution of cancer and a multi-stage theory of carcinogenesis. Br. J. Cancer.

[269] Fanali C., Lucchetti D., Farina M., Corbi M., Cufino V., Cittadini A., Sgambato A. (2014). Cancer stem cells in colorectal cancer from pathogenesis to therapy: Controversies and perspectives. World J. Gastroenterol.

[270] Trosko J.E. (2014). Induction of iPS cells and of cancer stem cells: The stem cell or reprogramming hypothesis of cancer?. Anat. Rec.

[271] Agathocleous M., Harris W.A. (2013). Metabolism in physiological cell proliferation and differentiation. Trends Cell Biol.

[272] Smith-Vikos T. (2012). A report of the James Watson lecture at Yale University. Yale J. Biol. Med.

[273] Pierce G.B. (1974). Neoplasms, differentiations and mutations. Am. J. Pathol.

[274] DeBerardinis R.J., Thompson C.B. (2012). Cellular metabolism and disease: What do metabolic outliers teach us?. Cell.

[275] Santin G., Paulis M., Vezzoni P., Pacchiana G., Bottiroli G., Croce A.C. (2013). Autofluorescence properties of murine embryonic stem cells during spontaneous differentiation phases. Lasers Surg. Med.

[276] Warburg O., Gawehn K., Geissler A.W., Lorenz S. (1960). On the transformation of embryonal metabolism into cancer metabolism. Hoppe. Seylers. Z. Physiol. Chem.

[277] Fliedner S.M., Kaludercic N., Jiang X.S., Hansikova H., Hajkova Z., Sladkova J., Limpuangthip A., Backlund P.S., Wesley R., Martiniova L. (2012). Warburg effect’s manifestation in aggressive pheochromocytomas and paragangliomas: Insights from a mouse cell model applied to human tumor tissue. PLoS One.

[278] Seppet E., Gruno M., Peetsalu A., Gizatullina Z., Nguyen H.P., Vielhaber S., Wussling M.H., Trumbeckaite S., Arandarcikaite O., Jerzembeck D. (2009). Mitochondria and energetic depression in cell pathophysiology. Int. J. Mol. Sci.

[279] Xu T., Zhao J., Hu P., Dong Z., Li J., Zhang H., Yin D., Zhao Q. (2014). Pentachlorophenol exposure causes Warburg-like effects in zebrafish embryos at gastrulation stage. Toxicol. Appl. Pharmacol.

[280] Campos B., Gal Z., Baader A. (2014). Aberrant self-renewal and quiescence contribute to the aggressiveness of glioblastoma. J. Pathol.

[281] Jiang Y., Zhou X., Chen X., Yang G., Wang Q., Rao K., Xiong W., Yuan J. (2011). Benzo(a)pyrene induced mitochondrial dysfunction and cell death in p53-null Hep3B Cells. Mutat. Res.

[282] Pavanello S., Dioni L., Hoxha M., Fedeli U., Mielzynska-Svach D., Baccarelli A.A. (2013). Mitochondrial DNA copy number and exposure to polycyclic aromatic hydrocarbons. Cancer Epidemiol. Biomark. Prev.

[283] Xie Y., Zhong C., Zeng M., Guan L., Luo L. (2013). Effect of hexavalent chromium on electron leakage of respiratory chain in mitochondria isolated from rat liver. Cell Physiol. Biochem.

[284] Liu G., Cheresh P., Kamp D.W. (2013). Molecular basis of asbestos-induced lung disease. Annu. Rev. Pathol.

